# Divergent camptothecin biosynthetic pathway in *Ophiorrhiza pumila*

**DOI:** 10.1186/s12915-021-01051-y

**Published:** 2021-06-16

**Authors:** Mengquan Yang, Qiang Wang, Yining Liu, Xiaolong Hao, Can Wang, Yuchen Liang, Jianbo Chen, Youli Xiao, Guoyin Kai

**Affiliations:** 1 Laboratory of Medicinal Plant Biotechnology, College of Pharmacy, Zhejiang Chinese Medical University, Hangzhou, 310053 Zhejiang China; 2 CAS Key Laboratory of Synthetic Biology, CAS Center for Excellence in Molecular Plant Sciences, Core Facility Centre, Institute of Plant Physiology and Ecology, Chinese Academy of Sciences, Shanghai, 200032 China; 3 Institute of Plant Biotechnology, School of Life Sciences, Shanghai Normal University, Shanghai, 200234 China

**Keywords:** Biosynthesis, Camptothecin, In vivo labelling, *Ophiorrhiza pumila*, Strictosidine

## Abstract

**Background:**

The anticancer drug camptothecin (CPT), first isolated from *Camptotheca acuminata*, was subsequently discovered in unrelated plants, including *Ophiorrhiza pumila*. Unlike known monoterpene indole alkaloids, CPT in *C. acuminata* is biosynthesized via the key intermediate strictosidinic acid, but how *O. pumila* synthesizes CPT has not been determined.

**Results:**

In this study, we used nontargeted metabolite profiling to show that 3*α*-(*S*)-strictosidine and 3-(*S*), 21-(*S*)-strictosidinic acid coexist in *O. pumila*. After identifying the enzymes *Op*LAMT, *Op*SLS, and *Op*STR as participants in CPT biosynthesis, we compared these enzymes to their homologues from two other representative CPT-producing plants, *C. acuminata* and *Nothapodytes nimmoniana*, to elucidate their phylogenetic relationship. Finally, using labelled intermediates to resolve the CPT biosynthesis pathway in *O. pumila*, we showed that 3*α*-(*S*)-strictosidine, not 3-(*S*), 21-(*S*)-strictosidinic acid, is the exclusive intermediate in CPT biosynthesis.

**Conclusions:**

In our study, we found that *O. pumila*, another representative CPT-producing plant, exhibits metabolite diversity in its central intermediates consisting of both 3-(*S*), 21-(*S*)-strictosidinic acid and 3*α*-(*S*)-strictosidine and utilizes 3*α*-(*S*)-strictosidine as the exclusive intermediate in the CPT biosynthetic pathway, which differs from *C. acuminata*. Our results show that enzymes likely to be involved in CPT biosynthesis in *O. pumila*, *C. acuminata*, and *N. nimmoniana* have evolved divergently. Overall, our new data regarding CPT biosynthesis in *O. pumila* suggest evolutionary divergence in CPT-producing plants. These results shed new light on CPT biosynthesis and pave the way towards its industrial production through enzymatic or metabolic engineering approaches.

**Supplementary Information:**

The online version contains supplementary material available at 10.1186/s12915-021-01051-y.

## Background

**Fig. 1:**
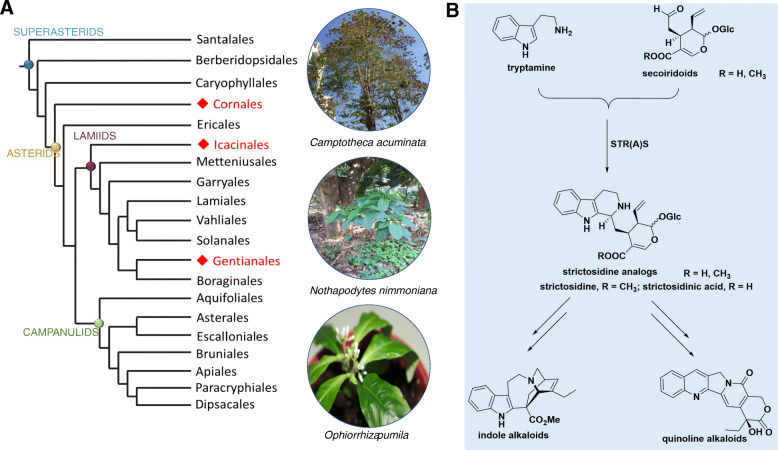
Distribution of CPT in the plant kingdom and possible biosynthesis of alkaloids through strictosidine or strictosidinic acid. **a** Phylogenetic tree of the major lineages of land plants summarizing the known taxonomic distribution of CPT-producing species. Orders that contain at least one CPT-producing species are indicated with red diamonds. The three CPT-producing plants used in this study are shown on the right and belong to the Cornales (*C. acuminata*), Icacinales (*N. nimmoniana*), and Gentianales (*O. pumila*) orders. **b** The biosynthesis of monoterpene indole alkaloids (MIAs) through strictosidine or strictosidinic acid derived from tryptamine and secoiridoids. STR, strictosidine synthase; STRAS, strictosidinic acid synthase

The alkaloid camptothecin (CPT) was first isolated in 1966 from the bark of the tree *Camptotheca acuminata* (“Xi-Shu” in Chinese, which translates to happy tree). *C. acuminata* is a deciduous tree native to southern China that is extensively used in traditional Chinese medicine (TCM) [1]. CPT chemotype also appears sporadically in multiple taxa within the superasterids, with a total of 43 plant species [2]. Of those, *C. acuminata*, *Nothapodytes nimmoniana*, and *Ophiorrhiza pumila* are the three main representative CPT-producing plants (Fig. 1a). In 1994, the US Food and Drug Administration (FDA) approved the therapeutic use of two well-known antitumour CPT derivatives, irinotecan and topotecan, which inhibit the replication, growth, and reproduction of cancer cells by inhibiting DNA topoisomerase I [3]. The clinical applications of these two CPT-derived drugs to the treatment of cancer have greatly increased demand, raising issues about the sustainable production of CPT [4]. Because chemical synthesis of CPT on an industrial scale is hindered by the complexity of its unique pentacyclic pyrroloquinoline scaffold, the sourcing of CPT still depends heavily on extraction from its resource plants [5, 6]. However, the few plants that naturally produce CPT grow slowly and will not meet this increasing market demand, necessitating an alternative approach to raise CPT production [6, 7]. One such promising method involves the introduction of key biosynthetic genes or regulators through metabolic engineering or the reconstruction of the CPT biosynthetic pathway in heterologous microbial systems by synthetic biology. This method will require the dissection and thorough understanding of CPT biosynthesis, which is currently lacking [7–11].

More than 2000 monoterpene indole alkaloids (MIAs) are thought to originate from the common intermediate strictosidine, which separates the MIA biosynthetic pathway into prestrictosidine and poststrictosidine pathways or steps (Fig. 1b) [12–14]. In plants, the biosynthesis of strictosidine has been well studied [6, 15–17]. The methylerythritol 4-phosphate (MEP) and mevalonic acid (MVA) pathways provide basic terpene precursors such as isopentenyl pyrophosphate (IPP) to form secologanin via the iridoid pathway [6]. The biosynthesis of secologanin involves the successive action of the enzymes geranyl diphosphate synthase (GPS), geraniol synthase (GE), geraniol 10-hydroxylase (G10H), 10-hydroxygeraniol oxidoreductase (10-HGO), iridoid synthase (IS), iridoid oxidase (IO), 7-deoxyloganetic acid UDP-glucosyltransferase (7-DLGT), 7-deoxyloganic acid hydroxylase (7-DLH, catalyzed by CYP72A224), secologanin synthase (SLS), and cytochrome P450 reductase (CPR), finally forming secologanin [14]. In the shikimate pathway, several enzymes participate in multistep reactions from chorismic acid to tryptamine, which is another precursor for CPT biosynthesis. These enzymes include anthranilate synthase (ASA), phosphoribosyl diphosphate anthranilate transferase (PRT), tryptophan synthase α (TSA), tryptophan synthase β (TSB), and tryptophan decarboxylase (TDC). TDC is considered to be a rate-limiting enzyme in CPT biosynthetic pathway [18]. Subsequently, tryptamine and secologanin are condensed into strictosidine by strictosidine synthase (STR). However, multiple enzymatic steps are still missing from our understanding of the poststrictosidine pathway, and only a few of the metabolite intermediates in the CPT biosynthetic pathway, such as pumiloside and deoxypumiloside, have been identified [19, 20].

Based on our current understanding of CPT biosynthesis, strictosidine is commonly considered the key intermediate in the biosynthesis of MIAs such as vinblastine and vincristine. However, although CPT belongs to the MIA family, no strictosidine was detected in the CPT-producing plant *C. acuminata* [15]. Furthermore, loganic acid, secologanic acid, and strictosidinic acid were all detected by metabolite profiling, whereas loganin, secologanin, and strictosidine were all undetectable in *C. acuminata*, leading to the conclusion that strictosidinic acid was the sole intermediate for CPT biosynthesis (Fig. 1b) [15]. In contrast, in *N. nimmoniana*, only secologanin was detected [21], indicating that strictosidine, and not strictosidinic acid, might be a key biosynthetic intermediate. In the case of *O. pumila*, independent metabolic profiling studies identified a strictosidinic backbone as a precursor but did not agree on the exact form, with one reporting strictosidinic acid and the other strictosidine [22, 23]. A bifunctional secologanin synthase (SLS) that catalyses the reaction of loganin (or loganic acid) to form secologanin (or secologanic acid) was characterized in *C. acuminata* [16]. However, the enzyme that converts secologanic acid and tryptamine into strictosidinic acid has not been identified or characterized in *C. acuminata*, *N. nimmoniana*, or *O. pumila*. Compared to the woody plants *C. acuminata* and *N. nimmoniana*, the herbaceous plant *O. pumila* (from the genus *Ophiorrhiza*) offers a number of advantages, including a shorter generation time and easier genetic transformation. *O. pumila* would therefore be an excellent system for metabolic engineering research into CPT biosynthesis [24]. However, it remains unclear which intermediate (strictosidine or strictosidinic acid) is most likely to take part in the CPT biosynthesis pathway in *O. pumila*.

To analyse CPT biosynthesis in *O. pumila*, we carried out nontargeted metabolite profiling and feeding experiments with deuterium-labelled tryptophan. Our results demonstrated the detection of both strictosidine and strictosidinic acid. Functional gene analyses and in vitro biochemical characterization further indicated that the *O. pumila* enzymes *Op*LAMT, *Op*SLS, and *Op*STR participated in the biosynthesis of strictosidine and strictosidinic acid. Feeding experiments of *O. pumila* with d_4_-strictosidine and d_4_-strictosidinic acid suggested that strictosidine, rather than strictosidinic acid, was the exclusive intermediate involved in CPT biosynthesis. Our results demonstrate that the biosynthesis of CPT in *O. pumila* mainly recruits strictosidine and not strictosidinic acid, which is quite different from CPT biosynthesis in *C. acuminata*, suggesting divergence in their respective biosynthetic pathways.

## Results

### Two parallel pathways of CPT biosynthesis were proposed by metabolite profiling of intermediates in *O. pumila*

**Fig. 2:**
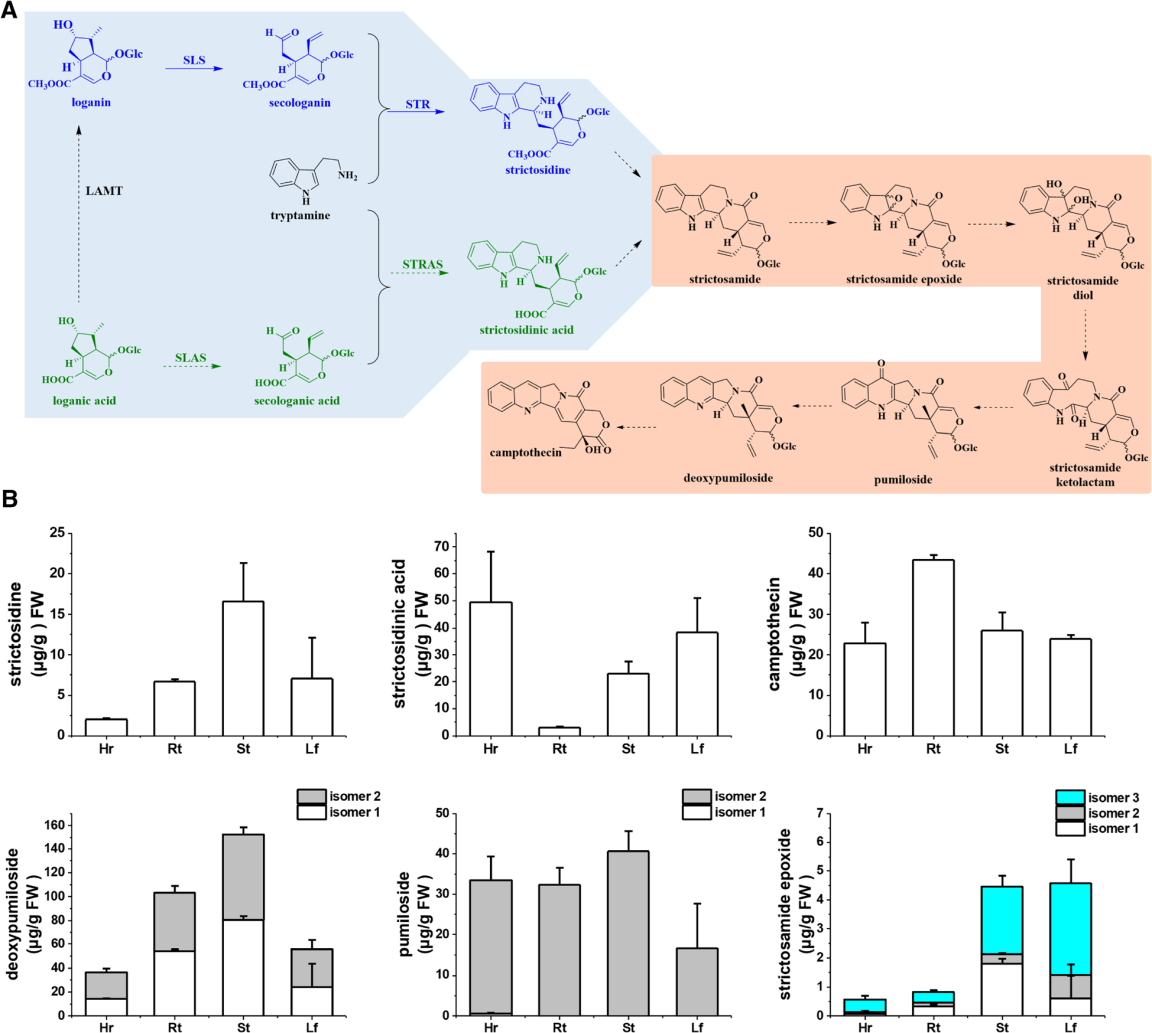
Two parallel pathways of CPT biosynthesis were proposed by metabolite profiling of intermediates in *O. pumila.* **a** Metabolites and identified enzymes involved in CPT biosynthesis in *O. pumila*. LAMT, loganic acid methyltransferase; SLAS, secologanic acid synthase; SLS, secologanin synthase; STR, strictosidine synthase; STRAS, strictosidinic acid synthase. Steps shaded in blue are part of the prestrictosidine pathway, while orange denotespoststrictosidine is the poststrictosidine pathway. **b** Tissue distribution profiles of proposed CPT pathway metabolites in *O. pumila* plants and hairy roots (4 replicates). Tissues were collected from wild-type plants grown on Gamborg B5 medium for 3 months, and methanol extracts were analysed using a 38.5-min gradient elution method for mass spectrometry (MS) detection. Multiple isomers were detected for deoxypumiloside, pumiloside, and strictosidine epoxide. Data are shown as the mean ± SD (*n* = 4) for the most abundant and quantifiable isomers. Hr, hairy root; Rt, root; St, stem; Lf, leaf

The distribution of the bioactive compound CPT varies across *O. pumila* tissues [23]. To assess the diversity and abundance of putative intermediates in the CPT biosynthetic pathway in *O. pumila*, we collected different tissues (leaves, stems, roots, and hairy roots) for metabolite profiling (Additional file 1: Fig. S1). We subjected total methanolic extracts to ultra-high-performance liquid chromatography (UHPLC) followed by mass spectrometry (MS) for untargeted metabolic analysis. We annotated 15 metabolites in the CPT biosynthetic pathway based on accurate mass measurements of positive ions and fragments observed in MS and MS/MS mass spectra (Fig. 2a and Additional file 1: Table S1) and confirmed them against the metabolite profiles detected in *C. acuminata* [15]. In this study, we detected both strictosidine and strictosidinic acid in *O. pumila* plant tissue and hairy root extracts. In addition, *O. pumila* plant tissues and hairy roots accumulated iridoids, loganic acid, loganin, and secologanic acid as well as secologanin. Therefore, in contrast to *C. acuminata*, both carboxylic acid derivatives (loganic acid, secologanic acid, and strictosidinic acid) and methyl ester derivatives (loganin, secologanin, and strictosidine) coexist in *O. pumila*. These results suggest that *O. pumila* might harbour two parallel pathways that produce CPT, in sharp contrast to *C. acuminata*, which uses carboxylic acid intermediates [15], and to the best-studied MIA-producing plants (e.g. the Madagascar periwinkle, *Catharanthus roseus*), which use methyl esters as intermediates [25, 26]. We also successfully detected additional metabolites in the CPT biosynthetic pathway previously reported in *O. pumila*, such as strictosamide, pumiloside, and deoxypumiloside [23].

To evaluate the possibility that two parallel CPT biosynthetic pathways might exist in *O. pumila*, we performed metabolite profiling and quantified the relative levels of all detectable metabolites as well as their isomers in extracts from four tissues (leaves, stems, roots, and hairy roots) (Fig. 2b). We identified several isomers with the same exact molecular masses and fragment ions as some of the metabolites we detected in *O. pumila*. We numbered these isomers according to their relative elution orders under the same liquid chromatography (LC) conditions (Additional file 1: Table S1). Strictosidine and strictosidinic acid coexisted in all tissues as a single isomer (Fig. 2b). This species differs from *C. acuminata*, which contains strictosidinic acid as three isomers and lacks any detectable strictosidine [15]. In *O. pumila*, the strictosidine content was highest in stems, while the strictosidinic acid content was highest in hairy roots (Fig. 2b). We detected two isomers of pumiloside (isomer 1 at very low levels) in hairy roots but only isomer 2 in other plant tissues (leaves, stems, roots) (Fig. 2b). We also detected two isomers of deoxypumiloside in all four tissues, with the highest levels in stems and roots (Fig. 2b). In addition, we identified three isomers for a compound we annotated as strictosamide epoxide, in accordance with *C. acuminata* [15] (Fig. 2b). Finally, we resolved a single CPT isomer, which we detected in all tissues, with the highest levels in roots (Fig. 2b). The presence of comparable amounts of both strictosidine and strictosidinic acid in all *O. pumila* tissues implies the possibility that parallel biosynthetic pathways for CPT might indeed exist, at least during the prestrictosidine stage. This raised the question of how these two key precursors might become incorporated into the poststrictosidine biosynthetic steps to generate CPT as the single and final product.

### Transcriptomic analysis and candidate gene identification in CPT biosynthesis

To further dissect the molecular basis of CPT biosynthesis, we performed deep sequencing and analysis of the transcriptome (RNA-seq) in the same *O. pumila* tissues used for extraction of metabolite intermediates. We followed the steps for transcriptome assembly, gene expression qualification, and gene annotation as described in our previous study [27].

Strictosidine is a common precursor in MIA biosynthesis (such as vinblastine in *C. roseus*). We thus hypothesized that CPT biosynthesis in *O. pumila* would also prefer strictosidine as a key intermediate. We therefore examined metabolite diversity in *O. pumila* in the prestrictosidine stage and performed bioinformatic analyses to identify the genes in *O. pumila* that might be involved in strictosidine biosynthesis by looking for putative *O. pumila* orthologues to the corresponding genes in *C. roseus* [26, 28, 29]. This analysis revealed that most of the *C. roseus* genes encoding enzymes in the MEP pathway for biosynthesis of strictosidine exhibited very high similarity (76–90% identity) to *O. pumila* genes, including *DXS*, *DXR*, *CMS*, *CMK*, *MCS*, *HDS*, *HDR*, *IPI*, *GPPS*, *G8H*, *GOR*, *ISY*, *IO*, *7DLGT*, *7DLH*, *LAMT*, and *SLS* (Additional file 1: Table S2). However, poststrictosidine genes encoding the enzymes responsible for catharanthine and tabersonine biosynthesis showed comparatively lower similarity (44–60%, with the exception of Redox1 at 71%, Additional file 1: Table S2), for example, *STR*, strictosidine β-d-glucosidase (*SGD*), geissoschizine synthase (*GS*), geissoschizine oxidase (*GO*), *Redox1*, *Redox2*, stemmadenine *O*-acetyltransferase (*SAT*), precondylocarpine acetate synthase (*PAS*), dehydroprecondylocarpine acetate synthase (*DPAS*), tabersonine synthase (*TS*), and catharanthine synthase (*CS*). In addition, these prestrictosidine genes from *C. acuminata* shared between 65 and 91% identity with *C. roseus* genes, with the exception of *LAMT* (54%), while poststrictosidine genes shared 38–60% identity (with the exception of *Redox1* (66%), Additional file 1: Table S2). Even though *O. pumila* and *C. roseus* belong to the same order (Gentianales), their poststrictosidine genes share low sequence similarity. This observation indicated that *O. pumila* and *C. roseus* diverged in the Gentianales order, at least in the context of their CPT biosynthetic pathway. The low sequence identity noted here between poststrictosidine genes in *C. roseus* and CPT-producing plants *O. pumila* and *C. acuminata* thus suggests a profound divergence in their respective biosynthetic pathways, adding confusion to our understanding of the poststrictosidine portion of their biosynthetic pathways.

### Expression patterns of prestrictosidine genes in *O. pumila*

Armed with the putative *O. pumila* orthologues for genes involved in strictosidine biosynthesis, we determined their expression levels from our RNA-seq dataset across our different tissues in *O. pumila*. We then clustered the genes involved in prestrictosidine to compare the resulting pattern with that of metabolite contents in the same tissues. The resulting heatmap revealed that genes from the later iridoid stage with high similarity to strictosidine biosynthetic genes, including five genes involved in iridoid biosynthesis (*IO*, *7DLGT*, *7DLH*, *LAMT*, and *SLS*) and *TDC*, were highly expressed in stems (Additional file 1: Fig. S2a). They displayed the highest expression in stems and lower expression in roots, followed by leaves, with hairy roots showing the lowest expression of these genes, which is consistent with the pattern of strictosidine content obtained by metabolite profiling (Fig. 2b and Additional file 1: Fig. S2a). Genes encoding proteins involved in the MEP pathway and in the early stage of iridoid biosynthesis were highly expressed in leaves and roots, respectively (Additional file 1: Fig. S2a). Based on the gene expression pattern, we speculate that genes related to the poststrictosidine pathway would be highly expressed in stems. However, biosynthetic genes involved in the prestrictosidine stage might exhibit different expression patterns from those involved in the poststrictosidine stage. The quantification of metabolites indicated that strictosidine accumulated to high levels in stems (Fig. 2b), whereas CPT accumulated to high levels in the root (Fig. 2b). Interestingly, it was found that the key intermediates (strictosidine, pumiloside, and deoxypumiloside) involved in the CPT biosynthetic pathway, except strictosidinic acid, accumulated to the highest levels in the stem, which indicates that the precursors of CPT are synthesized in the stem. There are three hypotheses to explain this contradiction. (1) Deoxypumiloside is transported into the roots and then converted into CPT in the roots by a series of enzymes. (2) CPT is also synthesized in the stems, which is consistent with other key intermediates (strictosidine, pumiloside, and deoxypumiloside), and then probably migrates from the stem to the root via transporters. (3) It is also possible that the very last steps for CPT biosynthesis are very active in the roots which leads to the high accumulation of CPT and low accumulation of intermediates, while these very last steps for CPT biosynthesis might be not very active in stems which results in high accumulation of intermediates and low accumulation of CPT. The identification of the relevant genes in *O. pumila* by comparable transcriptomic analyses opens the door to a more detailed exploration related to strictosidine biosynthesis.

### LAMTs, SLSs, and STRs from three genera of CPT-producing plants provide clues regarding CPT biosynthesis

To determine whether the gene encoding LAMT is present in the three representative CPT-producing species *C. acuminata*, *N. nimmoniana*, and *O. pumila*, we searched published transcriptomic databases for these three species. Indeed, the three species each code for one LAMT enzyme. Among them, *Ca*LAMT shared 53% identity with the *C. roseus* orthologue *Cr*LAMT (ABW38009.1), while *Op*LAMT shared 78% identity with *Cr*LAMT. Unfortunately, the sequence available for *Nn*LAMT (c51527_g1_i3) did not cover the full length of the gene [21]. However, *N. nimmoniana* accumulated the precursor secologanin [30], implying that *Nn*LAMT is a functional enzyme in vitro. We also characterized the cytochrome P450 gene *SLS* in *C. acuminata* and *C. roseus* and their encoded proteins. The bifunctional *Ca*SLS enzyme shared 65% identity with *Cr*SLS (AAA33106.1). *Nn*SLS (c54487_g1_i1) showed 76% identity with *Cr*SLS. *Op*SLS shared 83% identity with *Cr*SLS.

Is the sequence divergence between STR proteins restricted to a small domain of the protein? Comparing the STR sequences from these three species with that of *Cr*STR showed that *Ca*STR1 shared 38% identity with *Cr*STR, while *Op*STR showed 55% identity with *Cr*STR, and *Nn*STR shared 37% identity with *Cr*STR. We then aligned the protein sequences of LAMT, SLS, and STR (Additional file 1: Fig. S3), revealing many differences between STRs from the three species (Additional file 1: Table S3). This left unproven whether the observed sequence divergence might correlate with enzymatic activity.

### Phylogenetic analysis suggests greater evolutionary distance in CPT-producing plants

To further understand the evolutionary relationship between these proteins and their encoding genes, we next performed a molecular phylogenetic analysis of STRs from these three plant species, which revealed that they clustered in different clades (Additional file 1: Fig. S2b). This finding was in agreement with their relative positions in the general phylogenetic tree, which also indicated that they belong to different clades of flowering plants (Fig. 1a). In addition, we performed phylogenetic analyses of SLS and LAMT protein sequences deduced from available transcriptomic data. The six *Op*SLSs were divided into three clades and showed high similarity to *Nn*SLS, *Dc*SLS, and *Cr*SLSs (CYP72A1 and *Cr*CYP72C). In contrast, the *Ca*SLS proteins *Ca*CYP72A565 and *Ca*CYP72A610 formed a fourth clade (Additional file 1: Fig. S2c). The putative *Op*LAMT from *O. pumila* clustered away from other LAMTs, such as *Ca*LAMT and *Oe*LAMT, suggesting greater evolutionary distance (Additional file 1: Fig. S2d).

### In vitro biochemical characterization of *Op*LAMT, *Op*SLS, and *Op*STR

Following the identification of *O. pumila* genes encoding putative enzymes participating in CPT biosynthesis, the next step was to investigate their biocatalytic functions.

### *Op*LAMT shows loganic acid methyltransferase activity in vitro

**Fig. 3:**
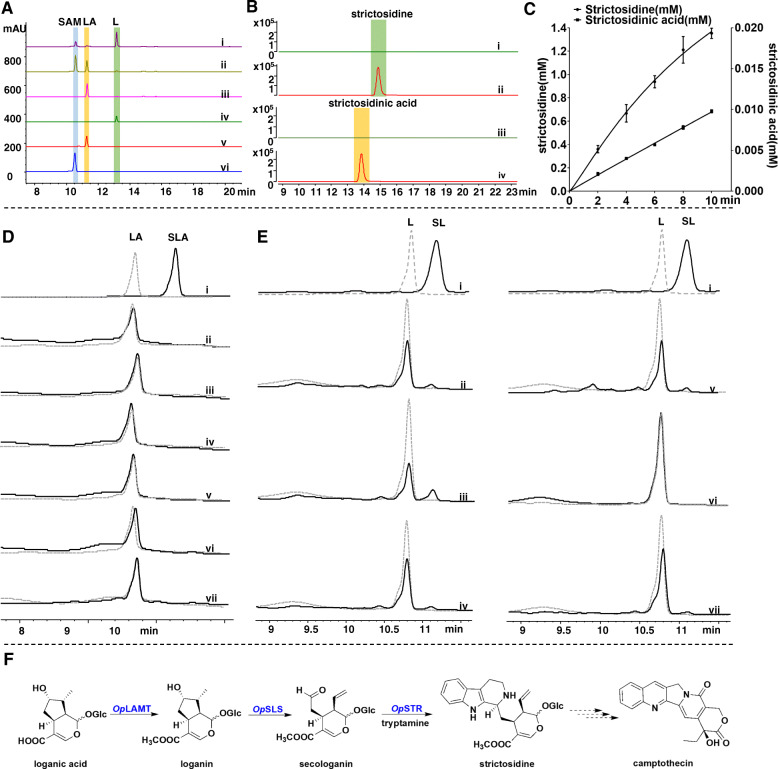
In vitro biochemical characterization of successive functional enzymes (*Op*LAMT, *Op*SLS, *Op*STR) involved in CPT biosynthesis in *O. pumila*. **a** Loganic acid methyltransferase HPLC assay with purified recombinant *Op*LAMT enzyme. (i) *Op*LAMT assay with loganic acid and SAM, (ii) boiled *Op*LAMT assay with loganic acid and SAM, (iii) *Op*LAMT assay with loganic acid, (iv) loganin (L) standard, (v) loganic acid (LA) standard, and (vi) *S*-adenosyl methionine (SAM) standard. **b** *Op*STR LC-MS assay with recombinant *Op*STR enzyme. Purified recombinant protein was assayed for strictosidine activity in reaction mixtures with secologanin/secologanic acid and tryptamine. (i) boiled STR assay with secologanin, (ii) STR assay with secologanin, (iii) boiled STR assay with secologanic acid, and (iv) STR assay with secologanic acid. **c** Time-course assay of STR activity. *Op*STR assay with secologanin (producing strictosidine) and secologanic acid (producing strictosidinic acid) (three repeats). The assays were quenched at 2 min, 4 min, 6 min, 8 min, and 10 min and then measured by LC-MS. Curve fitting was performed by GraphPad Prism 8. **d** Six *Op*SLS assays with loganic acid (LA)*.* The solid lines in black indicate samples from the microsome assay and secologanic acid standard (SLA). The dashed lines in grey indicate samples from the boiled microsome assay (control) and loganic acid standard. (i) Loganic acid and secologanic acid standards, (ii) *Op*SLS6, (iii) *Op*SLS5, (iv) *Op*SLS4, (v) *Op*SLS3, vi) *Op*SLS2, and (vii) *Op*SLS1. Wavelength, 254 nm. **e** Six *Op*SLSs assay with loganin (L). The solid lines in black indicate samples from the microsome assay and secologanin standard (SL). The dashed lines in grey indicate samples from the boiled microsome assay (control) and loganin standard. (i) Loganic acid and secologanic acid standards, (ii) *Op*SLS6, (iii) *Op*SLS5, (iv) *Op*SLS4, (v) *Op*SLS3, (vi) *Op*SLS2, and (vii) *Op*SLS1. Wavelength, 254 nm. **f** Summary of the CPT biosynthesis pathway in *O. pumila*. Loganic acid is converted into loganin in a reaction catalyzed by *Op*LAMT, and then loganin is converted into secologanin by *Op*SLSs. Secologanin and tryptamine are then condensed into strictosidine carried out by *Op*STR, which is involved in CPT biosynthesis in *O. pumila*

Unlike *C. roseus*, the intermediates loganin, secologanin, and strictosidine were not detected in *C. acuminata* during a previous metabolic study [15]. Their absence indicates that the relevant methyltransferases catalyzing the methylation of loganic acid, secologanic acid, and strictosidinic acid are either missing or have lost their function due to mutations in *C. acuminata*. Since we observed methyl ester intermediates in *O. pumila*, we suspected that a functional methyltransferase should exist to methylate the carboxylic acid intermediates. *Cr*LAMT demonstrated catalytic activity in *C. roseus* in a previous report [31]. Thus, we searched the transcriptomic database of *O. pumila* for *Cr*LAMT-like sequences and identified a single predicted *Op*LAMT with 78% identity to *Cr*LAMT (Additional file 1: Table S2). To evaluate its biochemical function, we cloned *Op*LAMT into the bacterial expression vector pET-30a and heterologously produced the protein in *Escherichia coli* BL21 (DE3) cells. The purified recombinant protein converted loganic acid and *S*-adenosyl methionine (SAM) into loganin (Fig. 3a). In addition, a microsome assay with secologanic acid as a substrate for recombinant *Op*LAMT revealed methylation of secologanic acid (Additional file 1: Fig. S4a). These results indicated that *Op*LAMT is a methyltransferase catalyzing both loganic acid and secologanic acid methylation into loganin and secologanin, respectively.

### *Op*STR shows promiscuous Pictet-Spengler reaction activity in vitro

Based on the results of metabolite profiling, we postulated that another STR-like enzyme might be involved in strictosidinic acid biosynthesis. However, a search of the *O. pumila* transcriptome database identified only one candidate, *Op*STR. To validate the phenomenon of the coexistence of strictosidine and strictosidinic acid in *O. pumila*, the activities of *Op*STR towards secologanin and secologanic acid were determined in recombinant enzyme assays. We purified recombinant *Op*STR to test its activity against secologanin and secologanic acid. Interestingly, LC-MS analysis indicated that *Op*STR can convert both secologanin and secologanic acid into 3*α*-(*S*)-strictosidine and 3-(*S*), 21-(*S*)-strictosidinic acid in vitro, respectively (Fig. 3b, compared with *Cr*STR). To compare the substrate specificity of *Op*STR, we performed a time-course assay (Fig. 3c). *Op*STR showed greater activity towards secologanin than towards secologanic acid as substrates. We quantified the products of the reactions against a standard curve for strictosidine and strictosidinic acid based on LC-MS peak integrations (Additional file 1: Fig. S5). We then fitted the data in GraphPad Prism 8 and calculated the resulting velocity (slope) ratio of assays towards secologanin and secologanic acid: *Op*STR displayed an activity towards secologanin 150 times higher than that for secologanic acid in the 10 min of the assay (Fig. 3c). In addition, we furtherly characterized the kinetic parameters of *Op*STR using LCMS by monitoring the production of strictosidine and strictosidinic acid. The results of the kinetic analysis are as follows: K_cat_/K_m_ = 8.23 min^−^1 mM^−1^ for secologanin and K_cat_/K_m_ = 0.00995 min^−^1 mM^−1^for secologanic acid (Additional file 1: Table S4). Here, we characterized *Op*STR showing promiscuous Pictet-Spengler reaction activity in vitro and supposed *Op*STR preferred secologanin as the substrate.

### *Op*SLS shows secologanin synthase activity only

To test the catalytic ability of our candidates in converting loganin and loganic acid, we cloned the six *Op*SLS genes (*Op*SLS1, CYP72A865; *Op*SLS2, CYP72A866; *Op*SLS3, CYP72A867; *Op*SLS4, CYP72A868; *Op*SLS5, CYP72A869; *Op*SLS6, CYP72A870) identified from the transcriptomic analysis into the yeast expression vector pESC-Leu and transformed the resulting constructs into yeast (strain WAT11). We extracted microsomes for activity assays: five *Op*SLS proteins (*Op*SLS1, *Op*SLS3, *Op*SLS4, *Op*SLS5, *Op*SLS6) exhibited secologanin synthase activity but not secologanic acid activity. However, *Op*SLS2 showed no detectable activity towards either secologanin or secologanic acid (Fig. 3d and e). These results demonstrate that the activities of *Op*SLS proteins are distinct from those of *Ca*SLSs and reflect their position within the phylogenetic tree (Additional file 1: Fig. S2c). Critically, these *Op*SLS assays also support a role for loganin and secologanin in CPT biosynthesis in *O. pumila*.

### STR assays from CPT-producing plants prove the validity of two parallel pathways in the plant kingdom

**Fig. 4:**
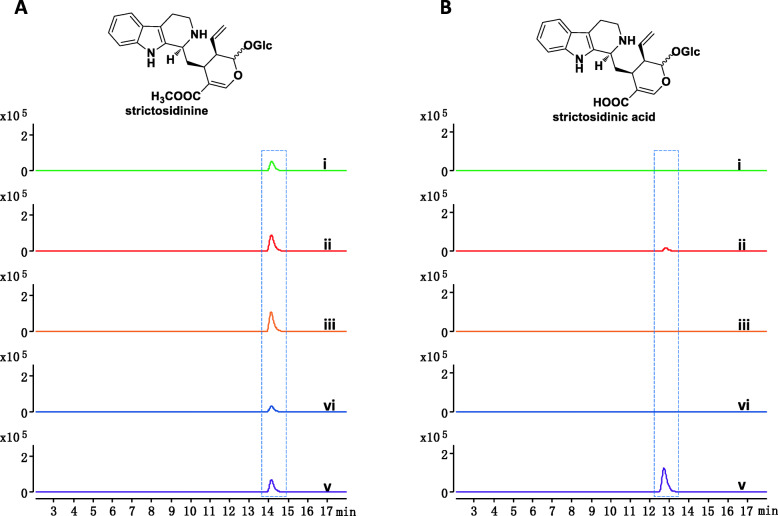
STR assays from CPT-producing plants prove the validity of two parallel pathways in the plant kingdom. **a** *Op*STR, *Ca*STR, and *Nn*STR activities towards secologanin. (i) *Ca*STR1, (ii) *Ca*STR2, (iii) *Ca*STR3, (iv) *Nn*STR, and (v) *Op*STR. The dashed box indicates the expected elution time of the STR product strictosidine. **b** *Op*STR, *Ca*STR, and *Nn*STR activities towards secologanic acid. (i) *Ca*STR1, (ii) *Ca*STR2, (iii) *Ca*STR3, (iv) *Nn*STR, and (v) *Op*STR. The dashed box indicates the expected elution time of the STR product strictosidinic acid

To better understand the differences in their CPT biosynthetic pathways, we cloned the *STR* genes from the three CPT-producing plants *C. acuminata*, *O. pumila*, and *N. nimmoniana* into the bacterial expression vector pET-30a and introduced the resulting constructs into *E. coli* BL21 (DE3). We then evaluated the enzymatic activities of *Op*STR, *Ca*STRs, and *Nn*STR towards secologanic acid and secologanin by LC-MS (Fig. 4). *Op*STR and *Ca*STR2 used both secologanin and secologanic acid activity as their substrates. In contrast, *Nn*STR, *Ca*STR1, and *Ca*STR3 displayed substrate specificity towards secologanin only. The observed specificities of STR enzymes indicated that strictosidine may play a major role in CPT biosynthesis, at least in *O. pumila* and *N. nimmoniana.* The activity exhibited by *Nn*STR is consistent with the metabolite profile of *N. nimmoniana*, as these plants contain only secologanin and no secologanic acid [30]. It remains unclear why the three *Ca*STRs each showed distinct enzymatic activity, even though their activity towards strictosidinic acid confirms the detection and isolation of strictosidinic acid in *C. acuminata* [15].

### In vivo labelling studies demonstrate that strictosidine is a key intermediate involved in CPT biosynthesis in *O. pumila*

**Fig. 5:**
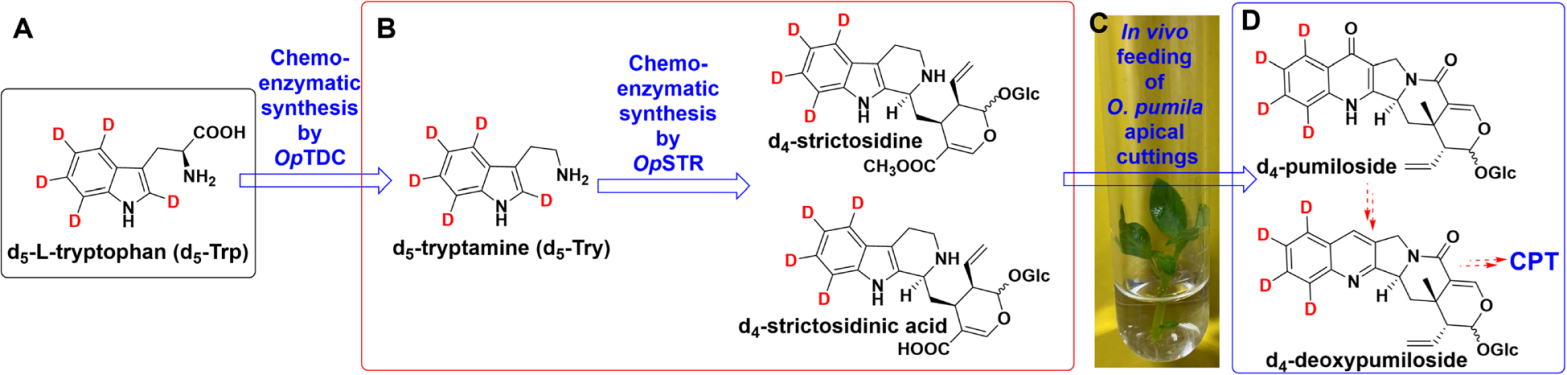
In vivo labelling to study the biosynthesis of *O. pumila* apical cuttings by feeding with deuterated metabolites. **a**, **b** Selected deuterium intermediates in the CPT biosynthesis pathway and their chemoenzymatic synthesis. **c** *O. pumila* apical cuttings were given the deuterated metabolites d_4_-strictosidine and d_4_-strictosidinic acid as feeding precursors. **d** Labelled poststrictosidine metabolite products with pentacyclic pyrroloquinoline scaffolds detected by in vivo feeding experiments

The detection of both the acid and methyl ester forms of the intermediates in the biosynthesis of strictosidinic acid and strictosidine in *O. pumila* lends support to our hypothesis that parallel biosynthetic pathways act in the prestrictosidine stage in this species. To further dissect metabolite flux in the poststrictosidine stage of CPT biosynthesis, key isotopically labelled metabolic intermediates are necessary for in vivo feeding studies. Starting from commercially available deuterated d_5_-l-tryptophan (d_5_-Trp), we chemoenzymatically synthesized three deuterium-labelled metabolites by using purified recombinant proteins (*Op*TDC, *Op*STR) (Fig. 5a, b and Additional file 1: Fig. S6). We purified the deuterium-labelled products and characterized them by LC-MS to confirm that they harboured the correct number of deuterium atoms due to labelling (Additional file 1: Fig. S7 and S8). With these synthesized deuterated intermediates, d_4_-strictosidine and d_4_-strictosidinic acid, as well as d_5_-l-tryptophan (d_5_-Trp), we conducted in vivo labelling studies by feeding *O. pumila* apical cuttings with the above-deuterated metabolites (Fig. 5c).

**Fig. 6:**
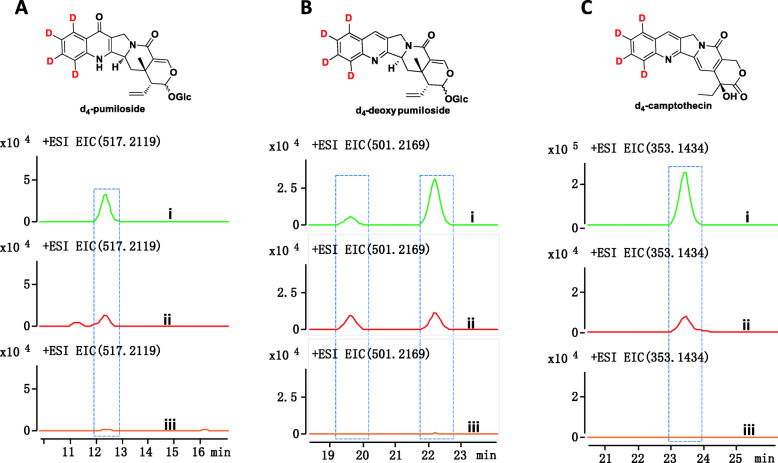
In vivo labelling studies demonstrate that strictosidine is a key intermediate involved in CPT biosynthesis in *O. pumila*. **a**–**c** Metabolites (d_5_-tryptophan [i], d_4_-strictosidine [ii], d_4_-strictosidinic acid [iii]) detected by LC-MS in extracts from feeding experiments with three different deuterium-labelled substrates: d_4_-pumiloside (**a**), d_4_-deoxypumiloside (**b**), and d_4_-camptothecin (**c**). Extracted ion chromatograms (EICs) of deuterium-labelled intermediates were compared

To trace the CPT biosynthetic pathway, we performed feeding experiments with each deuterium-labelled key intermediate (d_5_-tryptophan, d_4_-strictosidine, and d_4_-strictosidinic acid) provided individually at a concentration of 250 μM (Fig. 6). We incubated apical cuttings from wild-type plants in an aqueous solution with d_5_-tryptophan, d_4_-strictosidine, or d_4_-strictosidinic acid. After 45 days, we collected the stems and leaves for metabolite analysis via LC-MS. As expected, d_4_-strictosidine and d_4_-strictosidinic acid were detected in d_5_-tryptophan feeding experiment. Meanwhile, no d_4_-strictosidinic acid was detected in the d_4_-strictosidine feeding experiment, and no d_4_-strictosidine was detected in the d_4_-strictosidinic acid feeding experiment (Additional file 1: Fig. S9). It indicates that d_4_-strictosidine and d_4_-strictosidinic acid will not be converted into each other in vivo. Interestingly, we detected the poststrictosidine compounds pumiloside and deoxypumiloside, with a pentacyclic pyrroloquinoline scaffold [19], as well as CPT, in the extracts of plants incubated with d_4_-strictosidine but not with d_4_-strictosidinic acid compared to the extracts of plants incubated with d_5_-tryptophan (Fig. 6).

## Discussion

### *O. pumila* generates both carboxylic acids and methyl esters as proposed precursors in CPT biosynthesis

CPT was first identified in *C. acuminata* and later found in species belonging to unrelated angiosperm orders that were successively discovered [2], revealing an apparently random phylogenetic distribution of CPT production. Among known CPT-producing plants, the three representative species *N. nimmoniana*, *C. acuminata*, and *O. pumila* (Fig. 1b) exhibit chemical diversity in their CPT biosynthesis pathways. In many MIA-producing plants [14], such as *Apocynaceae* [32], *Rubiaceae* [7, 33], and *Icacinaceae* [30], strictosidine accumulates as a key intermediate in CPT biosynthesis and is then activated by conversion via the enzyme strictosidine β-d-glucosidase (SGD), whose unstable product strictosidine aglycone is rapidly converted into thousands of MIAs, such as vindoline, vinblastine, and vincristine, in *C. roseus* [25, 26, 34]. However, although *C. acuminata* produces MIAs, it was recently reported to accumulate only carboxylic acid precursors (loganic acid, secologanic acid, and strictosidinic acid). In agreement with this, strictosidinic acid was isolated from *C. acuminata* extracts [2, 15, 16, 35]. It is thought that strictosidine can also act as a key intermediate in CPT biosynthesis [22, 33]. However, *C. acuminata* appears to favour strictosidinic acid instead of strictosidine for CPT biosynthesis [15]. The coexistence of both carboxylic acids (loganic acid, secologanic acid, and strictosidinic acid) and methyl esters (loganin, secologanin, and strictosidine) in *O. pumila* was explained by global untargeted metabolite profiling in our study, which confirmed the results of previous studies that detected either strictosidinic acid or strictosidine in *O. pumila* [22, 23]. Interestingly, it was reported that only secologanin, the precursor of strictosidine, was detected in *N. nimmoniana* [21]. Even though both *C. acuminata* and *N. nimmoniana* are woody plants, they exhibit substantial differences in their key intermediates for CPT biosynthesis*.* In our study, we detected both strictosidine and strictosidinic acid in the leaves and roots of *N. nimmoniana* (Additional file 1: Fig. S10), indicating that *N. nimmoniana* and *O. pumila* accumulate both carboxylic acids and methyl esters as their proposed precursors during CPT biosynthesis. In addition, we identified loganic acid, loganin, secologanic acid, and secologanin in *O. pumila*. Collectively, these results strongly imply the evolution of at least two routes for CPT biosynthesis in CPT-producing plants. Carboxylic acid intermediates may be considered markers for the biosynthesis pathway seen in *C. acuminata*, while the coexistence of carboxylic acids and methyl esters in *O. pumila* and *N. nimmoniana* identifies another path to CPT. Here, we observed that strictosidinic acid accumulated to slightly higher levels than strictosidine in plants based on the quantification of metabolites. We hypothesize that 3*α*-(*S*)-strictosidine, but not 3-(*S*), 21-(*S*)-strictosidinic acid, is the key intermediate incorporated into CPT biosynthesis. Thus, 3-(*S*), 21-(*S*)-strictosidinic acid is probably the byproduct in the CPT biosynthetic pathway in *O. pumila*, resulting in high accumulation.

### Enzymatic evidence for the coexistence of carboxylic acids and methyl esters in *O. pumila*

To better understand the enzymatic basis of the chemical diversity underlying CPT biosynthesis, we performed transcriptome sequencing and analysis. In most MIA-producing species, such as *C. roseus*, secologanin and tryptamine are condensed into strictosidine; therefore, we searched and analysed the gene candidates for the prestrictosidine steps in CPT biosynthesis in *O. pumila*. As visualized by coexpression analysis, genes closely related to *Op*STR (*Op*IO, *Op*7DLGT, *Op*7DLH, *Op*LAMT, *Op*SLS, *Op*TDC) were highly expressed in stems and roots. Loganic acid *O*-methyltransferase (LAMT), secologanin synthase (SLS), and strictosidine synthase (STR) are involved in the formation of carboxylic acids and methyl esters, resulting in strictosidine and strictosidinic acid. We functionally characterized these enzymes in vitro (Fig. 3). We first demonstrated *Op*LAMT to be a promiscuous enzyme converting loganic acid and secologanic acid into loganin and secologanin, respectively (Fig. 3a and Additional file 1: Fig. S4a). By searching the *O. pumila* transcriptome database, we identified six *Op*SLS genes. Surprisingly, five out of the six *Op*SLS proteins showed activity towards loganin, although no *Op*SLS exhibited any activity towards loganic acid. These results indicate that either *Op*SLS activity towards loganic acid is too low to be detectable or that another *Op*SLS plays a role in converting loganic acid into secologanic acid. In an earlier study, the activities of *Op*STR were determined in recombinant enzyme assays [23]. They showed that *Op*STR converted tryptamine and secologanin into strictosidine. Here, we first determined that *Op*STR was a promiscuous enzyme capable of converting secologanic acid or secologanin into strictosidinic acid or strictosidine, respectively (Fig. 3b and c). The results from competition (Additional file 1: Fig. S4b) and time-course experiments (Fig. 3c) indicated that *Op*STR converted secologanin and tryptamine into strictosidine as its main product rather than catalyzing the formation of strictosidinic acid from secologanic acid and tryptamine (Additional file 1: Fig. S4b). This observation also implies that strictosidine may play a major role in CPT biosynthesis. Collectively, methyl ester intermediates (loganin, secologanin, strictosidine) and successive functional enzymes (*Op*LAMT, *Op*SLS, *Op*STR) are indeed involved in CPT biosynthesis in *O. pumila* (Fig. 3f). Based on their biochemical characterization, we postulate that strictosidine, not strictosidinic acid, is the main intermediate involved in CPT biosynthesis. To validate our hypothesis, we performed feeding experiments with proposed labelled precursors in vivo to determine their biotransformation profile.

### Strictosidine, not strictosidinic acid, is the central intermediate in CPT biosynthesis in *O. pumila*

Since both strictosidinic acid and strictosidine accumulated in all *O. pumila* tissues, we were unsure which might be involved in CPT biosynthesis in *O. pumila*. Satisfyingly, however, detectable amounts of labelled products with a pentacyclic pyrroloquinoline scaffold accumulated in tissues incubated with d_4_-strictosidine and d_5_-l-tryptophan (Fig. 6). However, we did not detect any labelled poststrictosidine compounds in extracts of tissues incubated with d_4_-strictosidinic acid (Fig. 6). These results are in sharp contrast with the observation that strictosidinic acid plays the role of a major precursor in CPT biosynthesis in *C. acuminata* but not strictosidine [15]*.* In addition, our metabolite analysis first showed that *N. nimmoniana* contained both strictosidine and strictosidinic acid (Additional file 1: Fig. S10), in contrast to only strictosidinic acid in *C. acuminata* [15]. These results indicate that both methyl ester derivatives and carboxylic acid derivatives coexist in *N. nimmoniana*, as in *O. pumila.* Collectively, our results indicate that strictosidine may play the same key role in *O. pumila* and *N. nimmoniana* and that strictosidinic acid fills in *C. acuminata*. Based on these observations, strictosidine, and not strictosidinic acid, is very likely a central intermediate in CPT biosynthesis in *O. pumila*, especially in the poststrictosidine stage. We further suggest that the CPT biosynthetic pathway in *O. pumila* is similar to most previously characterized MIA pathways, such as vinblastine biosynthesis in *C. roseus* and that *C. acuminata* differs from the more common CPT biosynthesis route. To further investigate the divergence among CPT-producing plants, we compared homologues across CPT-producing plants by biochemical assay.

### Evolution resulted in large differences in three representative CPT-producing plants

Most MIA-producing plants use strictosidine rather than strictosidinic acid as their central intermediate. We compared the genes involved in the CPT biosynthesis pathway with those of *C. roseus*, the best-studied MIA-producing plant. By comparing the enzymes involved in vincristine biosynthesis in *C. roseus* with those of *C. acuminata* and *O. pumila*, the enzymes involved in prestrictosidine all shared high identity. However, *O. pumila* appears to diverge in the poststrictosidine pathway according to low identity with the genes involved in the biosynthesis of specific MIAs such as vincristine (Additional file 1: Table S2), indicating that CPT-producing plants diverged from *C. roseus*. We hypothesize that the three CPT-producing plants probably utilize different precursors and show different enzymatic activities, indicative of independent evolution. Our in vivo deuterium-labelled metabolite feeding studies confirmed that strictosidine is the key intermediate in the poststrictosidine CPT biosynthetic pathway in *O. pumila*. The phylogenetic tree also supported the independent evolution of *N. nimmoniana*, *C. acuminata*, and *O. pumila* (Fig. 1b).

Why might *C. acuminata* produce strictosidinic acid as its intermediate for CPT biosynthesis? The enzymes involved in CPT biosynthesis in *C. acuminata* may provide some clues. Due to the absence of loganin, secologanin, and strictosidine, we postulate that *Ca*LAMT should not be a functional loganic acid methyltransferase. The recently characterized bifunctional *Ca*SLS [16] can convert both loganin and loganic acid with similar catalytic efficiency. According to the previous study [15], we postulate that *Ca*STRs probably show secologanic acid activity. However, we first discovered that strictosidine synthases (STRs) in *C. acuminata* mainly exhibited secologanin activity, and only one *Ca*STR showed detectable activity towards both secologanin and secologanic acid in our study (Fig. 4). The lack of methyl ester intermediates, combined with environmental pressures (biotic and abiotic stress), may have pushed *C. acuminata* to evolve a strictosidinic acid-dependent branch of the CPT biosynthetic pathway*.* At the same time, STRs from the three CPT-producing plants towards secologanic acid and secologanin show divergences in enzymatic activity (Fig. 4) and clustered into different clades in the phylogenetic tree (Additional file 1: Fig. S2b), which is consistent with the species tree (Fig. 1a). These observations indicate that the CPT biosynthesis pathway may have evolved divergently in flowering plants based on a comparison of the enzymes involved and the metabolite profiles in the three plant species. Thus, CPT biosynthesis in different CPT-producing plants likely utilizes two different routes. One is the traditional iridoid pathway, whereby loganin is converted into secologanin and then strictosidine by *Cr*SLS and *Cr*STR and later incorporated into MIA biosynthesis. The second route is the carboxylic acid pathway, in which loganin acid is converted into secologanic acid and then strictosidinic acid by *Ca*SLSs and *Ca*STRs, finally producing CPT through a series of bioconversion reactions in *C. acuminata* [15, 16]. In addition, *Nn*STR showed secologanin activity, which indicates strictosidine as the key intermediate incorporated into the CPT biosynthetic pathway in *O. pumila* and *N*. *nimmoniana*.

Resistance to CPT treatment is a hallmark of CPT-producing plants [17, 36, 37]. We therefore phylogenetically analysed DNA topoisomerase I sequences from several flowering plants. In CPT-producing and nonproducing species, we uncovered three key amino acid mutation sites related to CPT resistance (Additional file 1: Fig. S11). The endogenous biological function of CPT as a chemical defence molecule in host plants against biotic or abiotic insults is currently unknown. DNA topoisomerase I enzymes in these plants are resistant to the endogenous CPT they accumulate, presumably due to a mutation in CPT binding site (Additional file 1: Fig. S11) [17, 36, 37]. *O. pumila*, *Ophiorrhiza liukiuensis*, and *C. acuminata* share the N-to-S mutation. *N*. *nimmoniana* and *C. acuminata* share a specific N-to-K mutation. *O. pumila* and *O. liukiuensis* share a specific G-to-S mutation. Although the three plant species belong to three different orders (Gentianales (*O. pumila*), Icacinales (*N. nimmoniana*), and Nyssaceae (*C. acuminata*)), they display both divergence and some similarities regarding the genetic basis of their CPT resistance mechanism.

### Application in CPT production by metabolic engineering approaches

Medicinal plants accumulate very low levels of natural products, including various drugs with clinical applications, such as vinblastine and vincristine in *C. roseus* or CPT in *C. acuminata* and *O. pumila* [8, 11]. Here, our study provides a path towards improving CPT production in *Ophiorrhiza* species. *Op*LAMT is the key enzyme that controls the methylation of carboxylic acid intermediates. Together with *Op*STR, *Op*LAMT produces both strictosidinic acid and strictosidine. Strictosidine and strictosidinic acid coexist in *O. pumila*, and strictosidine production is a key factor in enhancing the production of CPT. Therefore, remodelling the pathway with a secologanin-specific STR and overexpression of LAMT might prove helpful to increase CPT production. In addition, our results provide some clues for the metabolic engineering of different CPT-producing plants in a microbe chassis.

## Conclusions

Most MIA-producing plants are thought to use strictosidine as the central intermediate in the biosynthesis of end-products, but the full CPT biosynthetic pathway in CPT-producing plants remains unclear. Surprisingly, it was found that *C. acuminata* uses strictosidinic acid instead of strictosidine as an intermediate for CPT biosynthesis. However, *O. pumila*, another representative CPT-producing plant, exhibits metabolite diversity in the central intermediates consisting of both 3-(*S*), 21-(*S*)-strictosidinic acid and 3*α*-(*S*)-strictosidine. We characterized a series of candidate genes in the prestrictosidine branch of the CPT pathway and compared them across the three representative CPT-producing plants *O. pumila*, *C. acuminata*, and *N. nimmoniana*. Our results show that enzymes likely to be involved in CPT biosynthesis in *O. pumila*, *C. acuminata*, and *N. nimmoniana* have evolved divergently. Overall, our new data about CPT biosynthesis in *O. pumila* suggest evolutionary divergence in CPT-producing plants. In addition, the promiscuity of LAMT and STR enzymes may pave the way towards the industrial production of CPT through enzymatic or metabolic engineering approaches.

## Methods

### Plant materials used in this study

*O. pumila* plants and hairy roots were obtained as reported previously [7]. Different tissues (leaves, stems, and roots) of 6-month-old *O. pumila* sterile seedlings and 3-month-old hairy roots grown on Gamborg’s B5 solid medium plates were collected for RNA-seq and metabolite profiling, respectively. In addition, apical cuttings of 6-month-old *O. pumila* plantlets were used in feeding experiments and cultured in Gamborg’s B5 liquid medium with deuterium-labelled substrates in 15 mL polypropylene round-bottom tubes (under 16 light, 8 h dark, 25 °C). After 45 days, the plant materials were used for metabolite extraction and LC-MS analysis.

### Nontargeted metabolites analysis

To analyse the metabolites of different plant tissues and hairy roots, we ground the above samples (including leaves, stems, roots, and hairy roots; Additional file 1: Fig. S1) to a fine powder under liquid nitrogen. We then added 500 μL of methanol to each sample (50 mg of powder); the methanol solution also contained 50 μM telmisartan as an internal standard. We vortexed the solution for 1 min, followed by extraction by ultrasonication at 4 °C for 30 min in an ice bath for 1 h. We then centrifuged all samples at 4 °C at 12,000*g* for 10 min, filtered the supernatants through a 0.22-μm filter membrane, and injected 1 μL of each sample into an Agilent 1290 UHPLC system coupled to an Agilent 6545 Q-TOF ESI high-resolution mass spectrometer (HRMS) for analysis. The column used for separation was an Agilent 300 Extend-C_18_ (4.6 × 150 mm, 3.5 μm) with the temperature set to 40 °C. Mobile phases A (H_2_O + 0.1% formic acid) and B (acetonitrile + 0.1% formic acid) were run in the following gradient programme at 0.3 mL/min: 0–1 min, 5% B; 1–3 min, 5–15% B; 3–11 min, 15–24% B; 11–18 min, 24–26% B; 18–30 min, 26–50% B; 30–33 min, 50–70% B; 33–34.5 min, 70–98% B; 34.5–38 min, 98% B; and 38–38.5 min, 98–5% B. The mass spectrometer was set to positive mode with a mass range between 70 and 1000 *m/z*. Other parameters were as follows: acquisition rate, 1.2 spectra/s; acquisition time, 833.3 ms/spectrum; gas temperature, 300 °C; drying gas, 6 L/min; nebulizer, 35 psig; vcap, 4000 V; fragmentor, 135 V; skimmer, 65 V; and oct 1 RF Vpp, 750 V. The collision energies used for MS fragmentation analysis were 20 V, 40 V, and 65 V.

### Transcriptome sequencing and bioinformatic analysis

We extracted total RNA from four different *O. pumila* tissues: leaves, stems, roots, and hairy roots. We prepared RNA sequencing libraries using the TIANGEN RNAprep Pure Plant Kit. We sequenced the resulting libraries on a NovaSeq 6000 platform according to the manufacturer’s instructions. Transcriptome assembly, gene quantification, and annotation were carried out as previously reported [27].

### Gene expression analysis

We determined the expression levels of most genes from the MEP pathway to the biosynthesis of strictosidine. The complete list of genes analysed here is as follows (further information and accession numbers are provided in Additional file 1: Table S2): 1-deoxy-d-xylulose-5-phosphate synthase (*DXS*), 1-deoxy-d-xylulose-5-phosphate reductoisomerase (*DXR*), 4-diphosphocytidylmethylerythritol 2-phosphate synthase (*CMS*), 4-diphosphocytidyl-2C-methyl-d-erythritol kinase (*CMK*), 2C-methyl-d-erythritol-2,4-cyclodiphosphate synthase (*MCS*), 1-hydroxy-2-methylbutenyl-4-diphosphate synthase (*HDS*), 1-hydroxy-2-methylbutenyl 4-diphosphate reductase (*HDR*), isopentenyl diphosphate isomerase (*IPI*), geraniol 8-hydroxylase (*G8H*), 8-hydroxygeraniol oxidoreductase (*GOR*), iridodial synthase (*ISY*), iridoid oxidase (*IO*), 7-deoxyloganetic acid glucosyltransferase (*7DLGT*), 7-deoxyloganic acid hydroxylase (*7DLH*), loganic acid *O*-methyltransferase (*LAMT*), secologanin synthase (*SLS*), and strictosidine synthase (*STR*). We visualized the gene expression levels and their hierarchical clustering as a heatmap (Additional file 1: Fig. S2a).

### Phylogenetic analyses

We downloaded the protein sequences for DNA topoisomerase I, STRs, SLSs, and LAMTs from the National Center for Biotechnology Information (NCBI) and the predicted protein sequences from the *O. pumila* transcriptome database for phylogenetic analyses. We aligned sequences with the help of ClustalW and generated the corresponding trees by the neighbour-joining method with the JTT model and bootstrap values set to 1,000 [38].

### Plasmid construction and enzymes preparation

We extracted the total RNA from plant tissues with the SPARKeasy RNA extraction kit (Sparkjade Science Co., Ltd.). Genes of interest were amplified by PCR from cDNA using the primers listed in Additional file 1: Table S5. *Escherichia coli* strain Top10 was used as the cloning host for plasmid construction, and *E. coli* BL21 (DE3) was used as the host for recombinant protein production. We introduced the plasmids pET30a-*Op*TDC, pET30a-*Op*LAMT, pET30a-*Op*STR, pET30a-*Ca*STR1, pET30a-*Ca*STR2, pET30a-*Ca*STR3, and pET30a-*Nn*STR individually into *E. coli* BL21(DE3). We inoculated 10 mL LB medium with single colonies for each construct, followed by cultivation at 37 °C for 12 h. We then transferred the culture into 1 L of fresh LB medium with kanamycin (50 mg/L) until the OD_600_ reached 0.6. For protein expression, we added 200 μM isopropyl-β-d-thiogalactoside (IPTG) to the cultures to induce protein production over 18 h at 16 °C. After collection by centrifugation, we suspended the cell pellets in 30 mL of lysis buffer (Sangon Biotech, B548117, consisting of 50 mM potassium phosphate buffer, pH 7.5, 100 mM NaCl and 5% glycerol) and lysed them via a Union-Biotech high-pressure homogenizer. After centrifugation at 20,000*g* for 40 min, we loaded the supernatant onto a column with Ni^2+^ resin. We used lysis buffer containing increasing concentrations of imidazole (25 mM, 50 mM, 100 mM, and 500 mM) to wash the column. Each fraction was sampled by SDS-PAGE analysis. We concentrated and desalted the target proteins on a PD-10 column and determined the protein concentration by Bradford assay using BSA to generate a standard curve.

To assess the activity of *Op*SLSs, we transformed the yeast expression vector pESC-Leu-SLSs into the WAT11 yeast strain. We selected transformants on a solid synthetic dropout medium lacking leucine and containing glucose as the carbon source. Yeast transformants were grown in 200 mL of synthetic dropout medium lacking leucine with glucose until they reached the logarithmic phase, at which point we harvested cells by centrifugation at 6000*g* for 5 min. We resuspended the cells in synthetic dropout medium lacking leucine with galactose as a carbon source to induce protein production for 36 h before collection. We prepared microsomes as previously reported [39].

### Enzymatic assays of *Op*LAMT and *Op*STR and HPLC-MS analysis

For *Op*LAMT, we performed a typical enzymatic assay in 100 μL aliquots of a reaction mixture containing 50 mM phosphate-buffered saline (PBS) buffer (pH 7.5), 1 mM loganic acid, and 1 mM *S*-adenosyl methionine (SAM) in the presence of loganic acid methyltransferase (LAMT) (1 mg/mL).

For *Op*STR, a typical enzymatic assay was carried out in 100 μL aliquots of a reaction mixture consisting of 50 mM PBS buffer (pH 7.5), 1 mM tryptamine, and 1 mM secologanin or secologanic acid in the presence of strictosidine synthase (STR) (1 mg/mL).

We incubated the reaction mixtures at 30 °C for 2 h and quenched the reactions with the addition of 100 μL of methanol and vortexing for 5 min. After centrifugation at 12,000*g* for 5 min and filtration, we used a 10-μL sample for LC-MS analysis. The column applied for analysis was an Agilent Eclipse plus C_18_ column (4.6 × 150 mm, 3.5 μm) on an Agilent 1260-6125+ LC-MS system with the temperature set at 35 °C. Mobile phases A (H_2_O + 0.1% formic acid) and B (acetonitrile) were run in the following gradient programme at 0.8 mL/min: 0–3 min, 5% B; 3–12 min, 5–30% B; 12–15 min, 30–95% B; 15–18 min, 95% B; 18–21 min, 95–5% B; and 21–24 min, 5% B. A 10-μL sample was injected for analysis. *Op*LAMT assays were monitored at 254 nm, and *Op*STR assays were monitored by the extracted ion chromatogram of the products.

### *Op*SLS microsome assay and HPLC analysis

We performed *Op*SLS microsome assays in 100 μL of the above-prepared microsomes containing 1 mM nicotinamide adenine dinucleotide phosphate (NADPH) and 1 mM specific substrate (loganin or loganic acid). We initiated the catalytic reaction through the addition of NADPH and incubated the reaction mixture at 30 °C. We quenched the reaction mixtures after 2 h with the addition of 100 μL of methanol. After the removal of the denatured proteins by centrifugation at 12,000*g* for 5 min, we analysed the supernatants by HPLC.

The column applied for analysis was a Phenomenex Luna C_18_(2) (4.6 × 250 mm, 5 μm) on an Agilent 1260 Infinity II system with the temperature set at 35 °C. Mobile phases A (H_2_O + 0.1% formic acid) and B (acetonitrile + 0.1% formic acid) were run in the following gradient programme at 0.8 mL/min: 0–3 min, 5% B; 3–12 min, 5–60% B; 12–15 min, 60–95% B; and 15–19 min, 95% B. A 10-μL sample was injected for analysis. *Op*SLS assays were monitored at 254 nm.

### Chemo-enzymatic synthesis of deuterium-labelled substrates

To trace the biosynthetic pathway of CPT, we performed a large-scale enzymatic reaction for deuterium-labelled product production. For d_5_-tryptamine production, we mixed 2 mM d_5_-tryptophan and 5 mM pyridoxal 5′-phosphate (PLP) in 30 mL *Op*TDC (2 mg/mL) solution at 30 °C for 10 h and concentrated the isolated product (d_5_-tryptamine) to dryness. For d_4_-strictosidine and d_4_-strictosidinic acid production, we mixed 2 mM purified d_5_-tryptamine and 5 mM secologanin or secologanic acid, respectively, in 10 mL at 30 °C until d_5_-tryptamine was completely consumed. d_4_-strictosidine and d_4_-strictosidinic acid were concentrated to dryness.

To check the purity of the deuterium-labelled product, we characterized the products by LC-MS to confirm the correct number of deuterium atoms incorporated (Additional file 1: Fig. S7 and S8).

### Feeding experiments and metabolite detection by LC-MS

We used deuterium-labelled substrates (d_5_-tryptophan, d_4_-strictosidine, and d_4_-strictosidinic acid) in the feeding experiment. We incubated the apical cuttings from plants grown on Gamborg’s B5 medium in an aqueous solution containing 250 μM d_5_-tryptophan, d_4_-strictosidine, and d_4_-strictosidinic acid. After 30 days, we collected the stems and leaves for metabolite analysis via LC-MS with the method mentioned in the “Nontargeted metabolite analysis” section.

## Supplementary Information

**Additional file 1: Fig. S1.** *O. pumila* plant materials were used for metabolite profiling. **Fig. S2.** Analysis of gene expression patterns and phylogenic analysis of STRs, SLSs, LAMTs enzymes in CPT biosynthesis. **Fig. S3.** Protein sequence alignments mentioned in this article. **Fig. S4.** *Op*LAMT assay with secologanic acid and *Op*STR competion expriments. **Fig. S5.** The standard curve of strictosidine and strictosidinic acid. **Fig. S6.** The SDS-PAGE gel of purified recombinant proteins used in the chemo-enzymatic synthesis of deuterium-labeled metabolites and biochemical assay. **Fig. S7.** Scheme of labeled substrates synthesis. **Fig. S8.** Chemoenzymatic synthesis of labeled substrates. **Fig. S9.** Detection of d_4_-strictosidinic acid and d_4_-strictosidine in the feeding experiments in *O. pumila*. **Fig. S10.** Metabolites detection of *N. nimmoniana* by LC-MS. **Fig. S11.** Phylogenetic relationship of DNA topoisomerase I in CPT-producing and non-producing species. **Table S1.** Relevant Compounds Detected in *O. pumila* plant and hairy root. **Table S2.** Identification of candidate CPT biosynthetic pathway genes in *O. pumila* as revealed by sequence identity with characterized genes from the pre-strictosidine biosynthetic pathways in *Catharanthus roseus*. **Table S3.** Identities and similarities among STRs from *C. acuminata*, *N. nimmoniana*, and *O. pumila* used in this work. **Table S4.** Kinetic parameters of *Op*STR towards secologanin and secologanic acid. **Table S5.** Primers list used in this study.

**Additional file 2:.** The original, uncropped SDS-PAGE of Fig. S6.

## Data Availability

The raw sequence data reported in this paper have been deposited in the Genome Sequence Archive in BIG Data Center, Beijing Institute of Genomics (BIG), Chinese Academy of Sciences, under accession number: CRA003143 [40]. The GenBank accession numbers for *Op*LAMT, *Op*SLS1, *Op*SLS2, *Op*SLS3, *Op*SLS4, *Op*SLS5, *Op*SLS6, *Ca*STR1, *Ca*STR2, and *Ca*STR3 are MT942677, MT942678, MT942679, MT942680, MT942681, MT942682, MT942683, MT942684, MT942685, and MT942686, respectively. Other data supporting the results in this study were shown in the Additional file.
