# Supplementary material for: Divergent camptothecin biosynthetic pathway in Ophiorrhiza pumila
Source: BMC Biol. 2021 Jun 16;19:122. doi: 10.1186/s12915-021-01051-y (PMC8207662; doi:10.1186/s12915-021-01051-y)
Supplement: Supplementary file 1 — Additional file 1: Fig. S1. O. pumila plant materials were used for metabolite profiling. Fig. S2. Analysis of gene expression patterns and phylogenic analysis of STRs, SLSs, LAMTs enzymes in CPT biosynthesis. Fig. S3. Protein sequence alignments mentioned in this article. Fig. S4. OpLAMT assay with secologanic acid and OpSTR competion expriments. Fig. S5. The standard curve of strictosidine and strictosidinic acid. Fig. S6. The SDS-PAGE gel of purified recombinant proteins used in the chemo-enzymatic synthesis of deuterium-labeled metabolites and biochemical assay. Fig. S7. Scheme of labeled substrates synthesis. Fig. S8. Chemoenzymatic synthesis of labeled substrates. Fig. S9. Detection of d4-strictosidinic acid and d4-strictosidine in the feeding experiments in O. pumila. Fig. S10. Metabolites detection of N. nimmoniana by LC-MS. Fig. S11. Phylogenetic relationship of DNA topoisomerase I in CPT-producing and non-producing species. Table S1. Relevant Compounds Detected in O. pumila plant and hairy root. Table S2. Identification of candidate CPT biosynthetic pathway genes in O. pumila as revealed by sequence identity with characterized genes from the pre-strictosidine biosynthetic pathways in Catharanthus roseus. Table S3. Identities and similarities among STRs from C. acuminata, N. nimmoniana, and O. pumila used in this work. Table S4. Kinetic parameters of OpSTR towards secologanin and secologanic acid. Table S5. Primers list used in this study. [file 12915_2021_1051_MOESM1_ESM.docx]

**Materials and Methods**

Commercial chemicals used in this work were listed in the below table. d_5_-tryptamine (indole-D5), d_4_-strictosidine (indole-D4) and d_4_-strictosidinic acid (indole-D4) were synthesized by large scale chemoenzymatic method.

| **Chemical** | **Source** | **Order No.** |
| --- | --- | --- |
| secologanin | Sigma Aldrich | 50741-5MG-F |
| secologanic acid | ChemFaces | CFN95028 |
| tryptophan | Aladdin | T118579-25g |
| tryptamine hydrochloride | Aladdin | T113731-25g |
| loganin | TargetMol | T2759 |
| Loganic acid | Sichuan Weikeqi Biological Technology Co., Ltd | wkq-00367 |
| L-tryptophan (indole-D5) | Cambridge Isotope Laboratories, Inc. | DLM-1092-PK |
| d_5_-tryptamine | Synthesized in this study |  |
| d_4_-strictosidine | Synthesized in this study |  |
| d_4_-strictosidinic acid | Synthesized in this study |  |

**
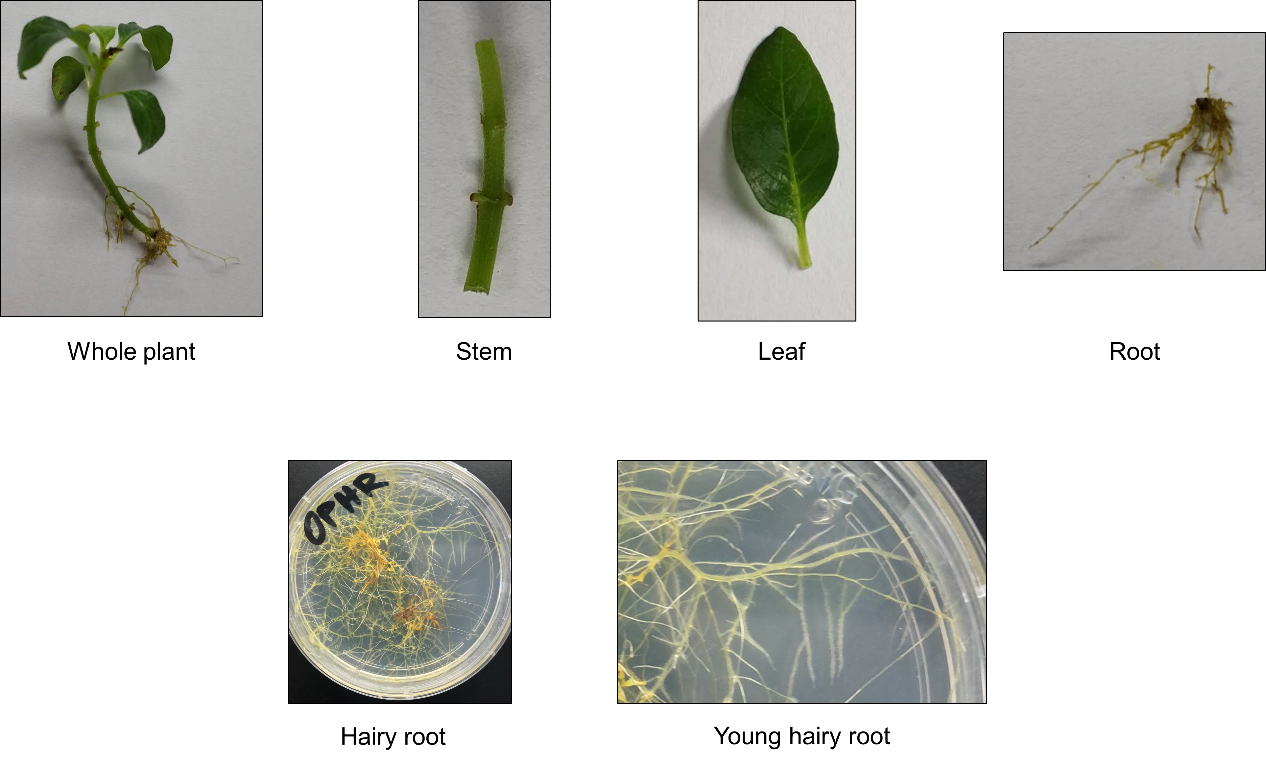
**

**Fig. S1. *O. pumila* plant materials were used for metabolite profiling.** The whole plant of *O. pumila* was cut to three different tissues (leaf, stem, and root). Hairy root as well as the plant were cultured on Gamborg's B5 Media in illumination incubator.

**Fig. S2. Analysis of gene expression patterns and phylogenic analysis of STRs, SLSs, LAMTs enzymes in CPT biosynthesis.**

**a** Heatmap visualization of the expression pattern of genes involved in the prestrictosidine pathway. Op_Hr, hairy root; Op_St, stem; Op_Lf, leaf; Op_Rt, root.

**b** Phylogenic analysis of strictosidine synthases (STRs) involved in indole alkaloid biosynthesis. CPT-producing plants are indicated with green stars.

**c** Six *Op*SLSs are divided into three clades (marked purple, light blue, pink).

**d** *Op*LAMT and *Ca*LAMT are distant in the relationship.

**A
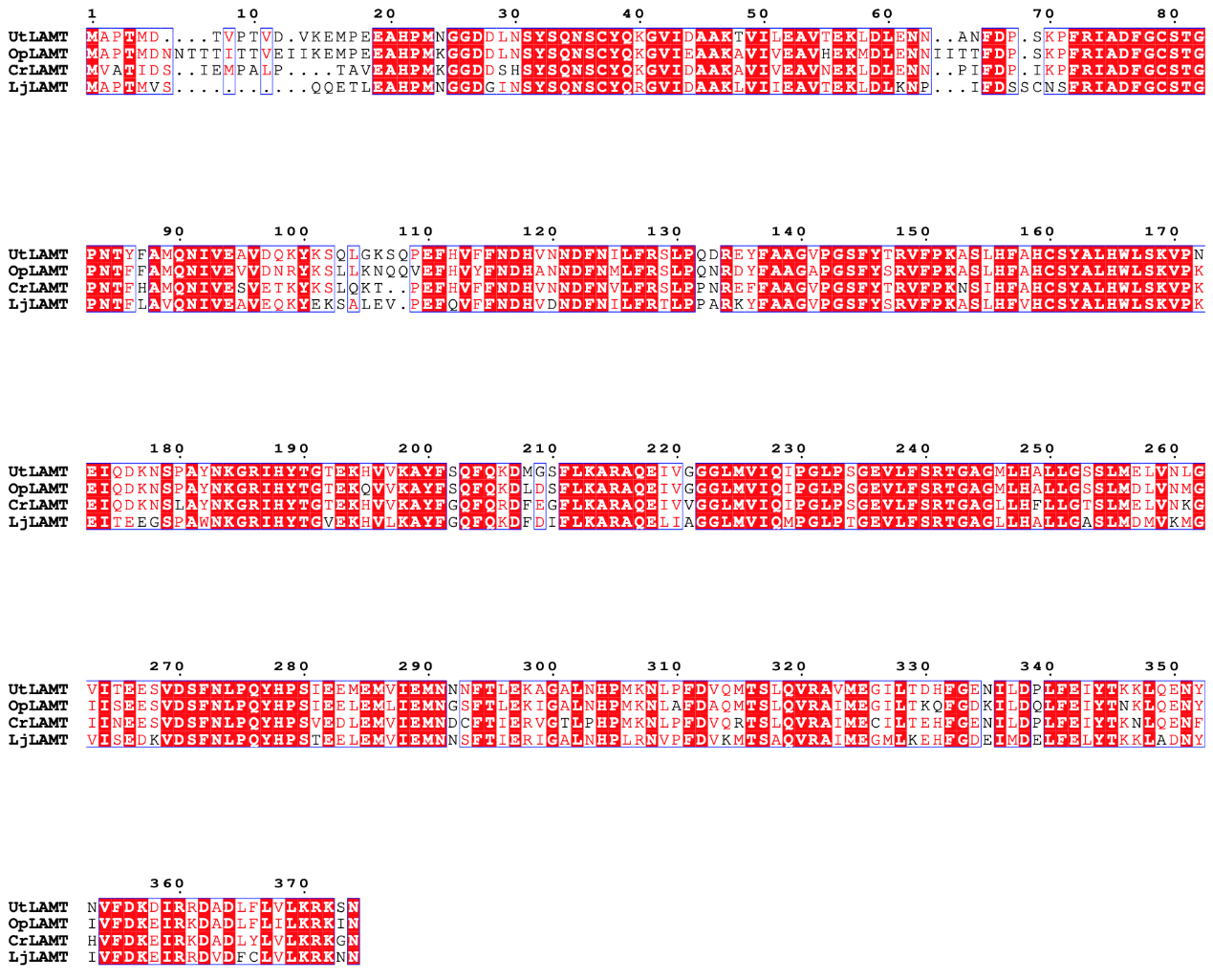
**

**Fig. S3. Protein sequence alignments mentioned in this article.**

**a** Amino acid sequence alignment of proteins from LAMTs.

**B**

**
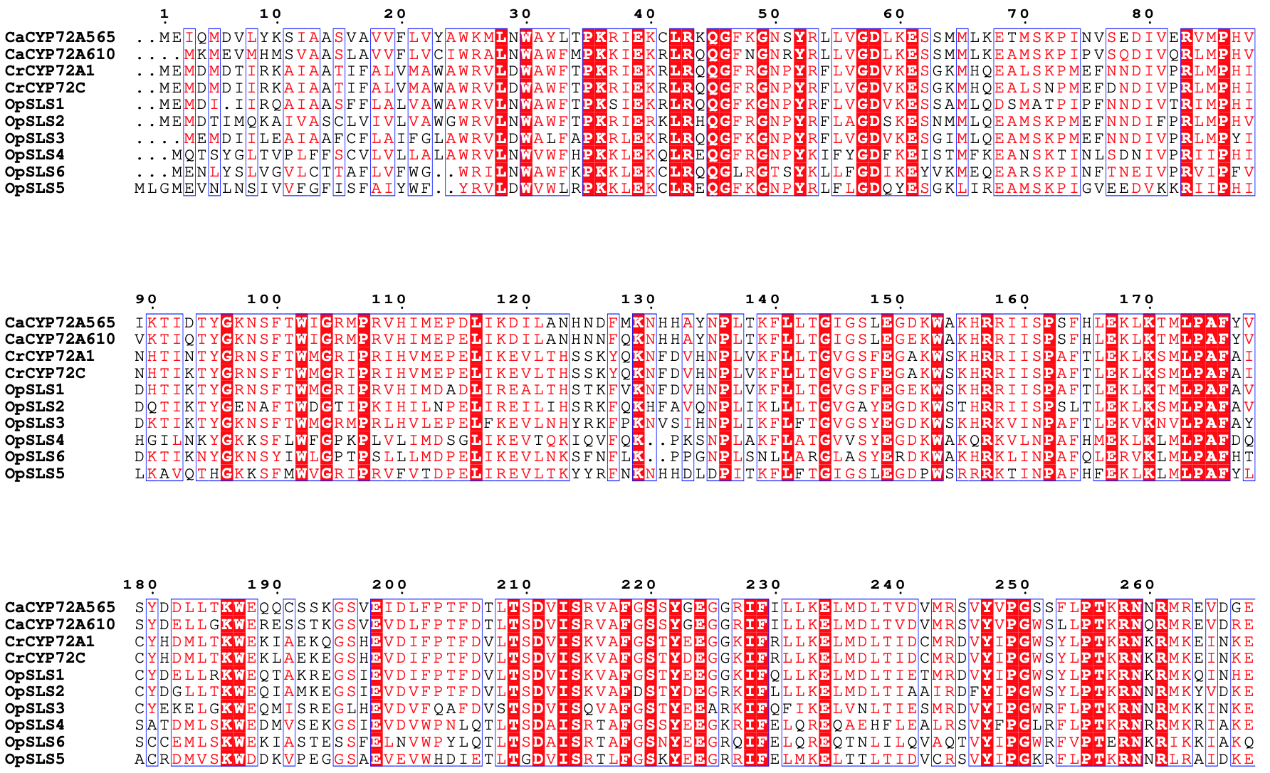

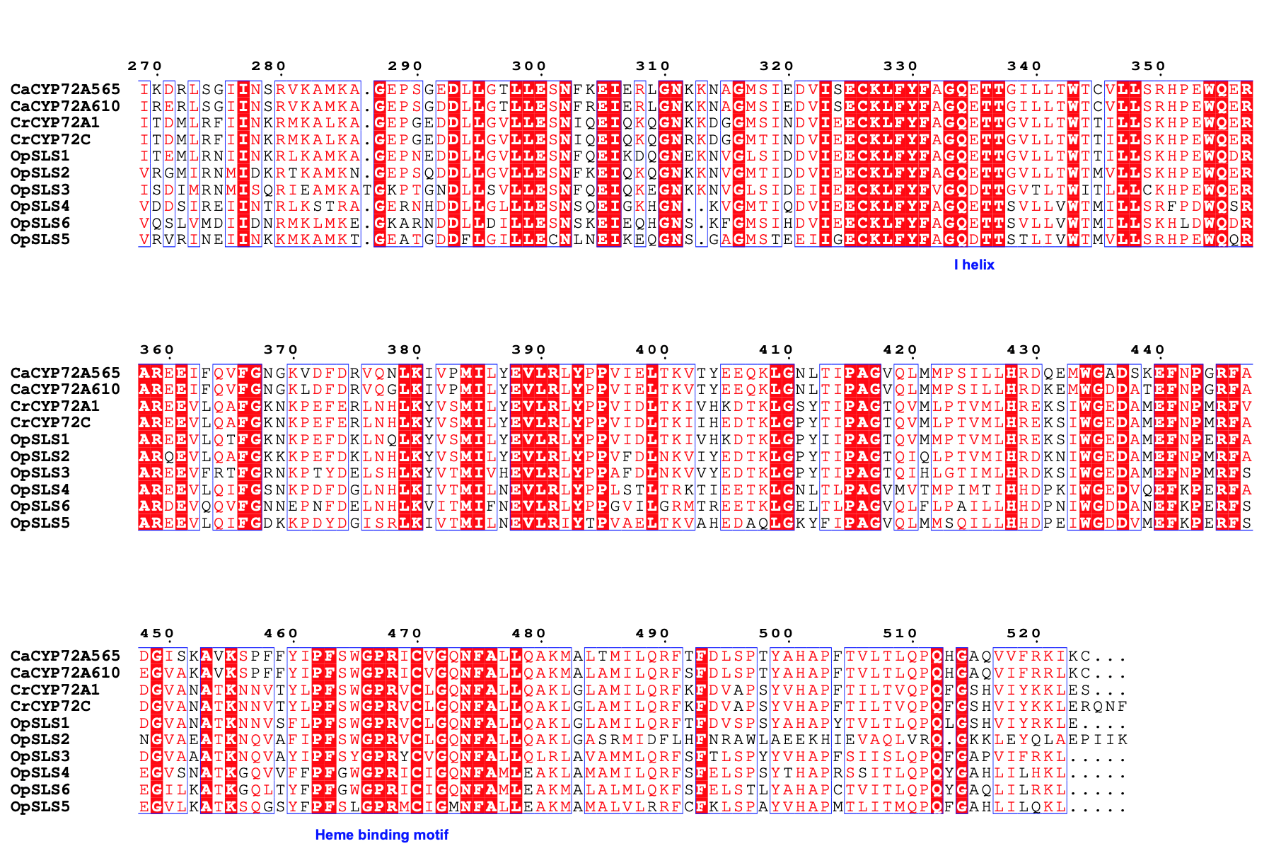
**

**Fig. S3. Protein sequence alignments mentioned in this article.**

b Amino acid sequence alignment of proteins from SLSs.

**C**


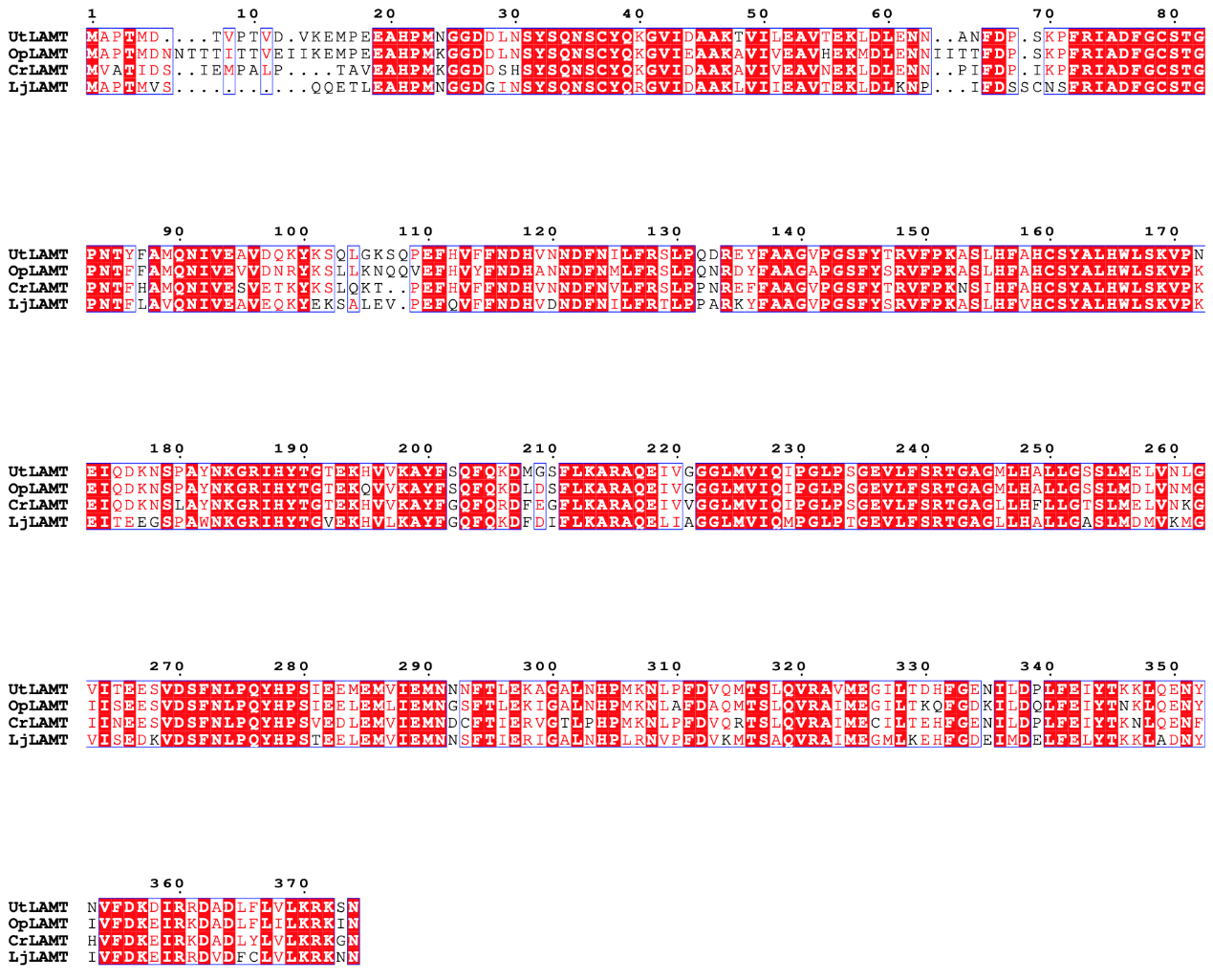


**Fig. S3. Protein sequence alignments mentioned in this article.**

**c** Amino acid sequence alignment of proteins from STRs.

**Fig. S4. a** *Op*LAMT assay with secologanic acid. The product (secologanin) was detected by LC-MS. (i) secologanic acid and SAM were mixed and supplied with *Op*LAMT. (ii) secologanic acid and SAM were mixed and supplied with boiled *Op*LAMT. **b** *Op*STR competion expriments. *Op*STR assay with 1 mM secologanic acid, 1mM secologanin and 1 mM tryptamine. (i) Extracted-ion chromatogram (EIC) of strictosidinic acid; (ii) Extracted-ion chromatogram (EIC) of strictosidine.


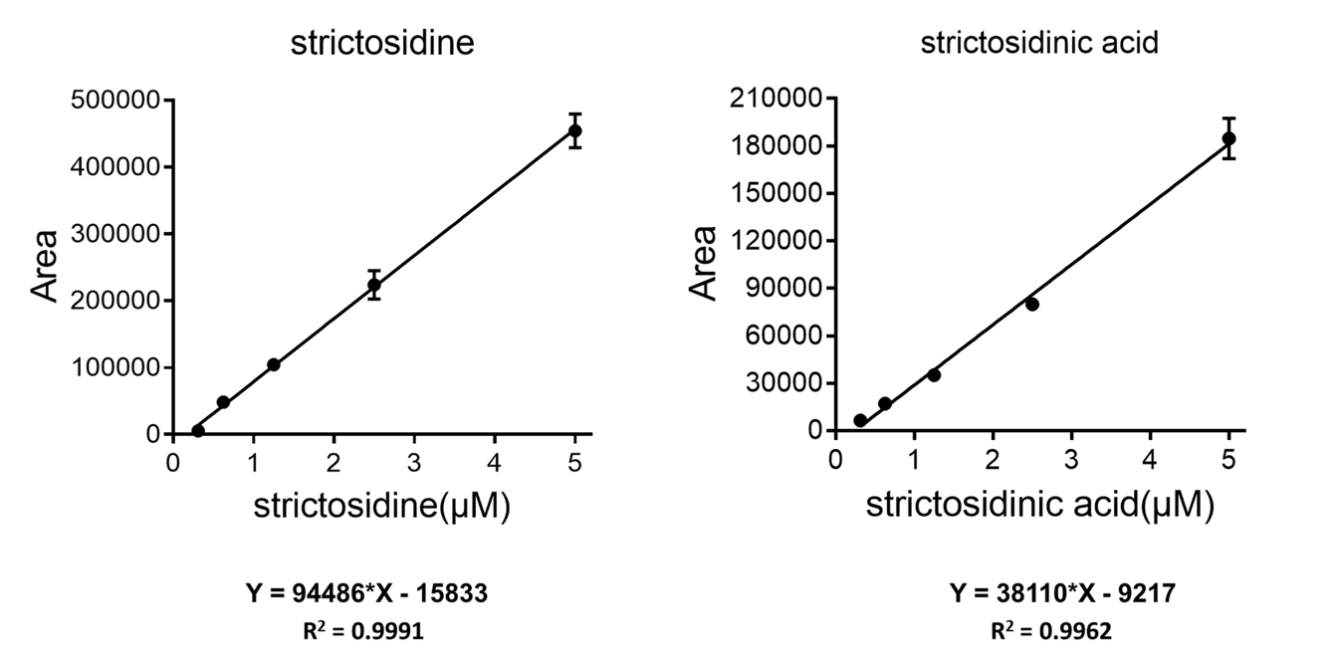


**Fig. S5. The standard curve of strictosidine and strictosidinic acid.** A series concentration of strictosidine and strictosidinic acid were detected by LC-MS (three replicates).

**Fig. S6. The SDS-PAGE gel of purified recombinant proteins used in the chemoenzymatic synthesis of deuterium-labelled metabolites and biochemical assay.**

**a** The SDS-PAGE gel of purified recombinant proteins *Op*TDC (left) and *Op*LAMT (right).

**b** The SDS-PAGE gel of purified recombinant proteins *Op*STR.

**Fig. S7. Scheme of labelled substrates synthesis.**

**a** d_5_-L-tryptophan was catalyzed by *Op*TDC into d_5_-tryptamine.

**b** d_5_-tryptamine and secologanic acid were catalyzed by *Op*STR into d_4_-strictosidinic acid.

**c** d_5_-tryptamine and secologanin were catalyzed by *Op*STR into d_4_-strictosidine.

**Fig. S8. Chemoenzymatic synthesis of labelled substrates.** w/o isotopic labelling compounds were compared by LC-MS. Purified recombinant proteins (*Op*TDC, *Op*STR) were used for producing isotopic labelling compounds (w/o isotopic labelling compounds were compared by LC-MS).

**a** Tryptamine (up) / d_5_-tryptamine (down); tryptamine, [M+H]^+^ = 161; d_5_-tryptamine, [M+H]^+^ = 166.

**b** Strictosidinic acid (up) / d_4_-strictosidinic acid (down) strictosidinic acid, [M+H]^+^ = 517; d_4_-strictosidinic acid, [M+H]^+^ = 521.

**c** Strictosidine (up) / d_4_-strictosidine (down); strictosidine, [M+H]^+^ = 531; d_4_-strictosidine, [M+H]^+^ = 535.


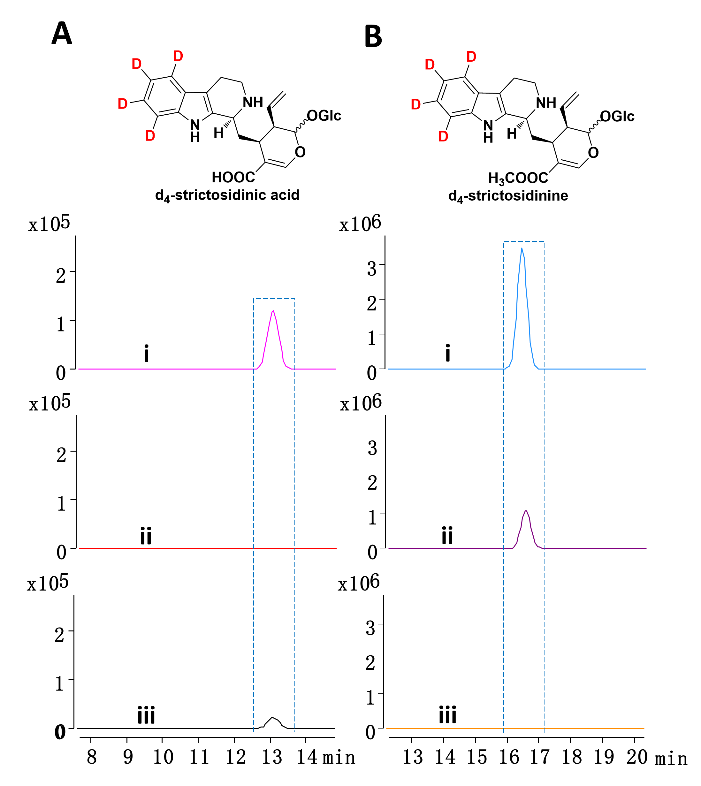


**Fig. S9. Detection of d_4_-strictosidinic acid and d_4_-strictosidine in the feeding experiments in *O. pumila*. a**-**b** Metabolites (d_5_-tryptophan [i], d_4_-strictosidine [ii], d_4_-strictosidinic acid [iii]) detected by LC-MS in extracts from feeding experiments with three different deuterium-labelled substrates: d_4_-strictosidinic acid (**a**) and d_4_-strictosidine (**b**). Extracted ion chromatograms (EICs) of deuterium-labelled intermediates were compared.

**Fig. S10. Metabolites detection of *N. nimmoniana* by LC-MS.** Extracts of leaves (i) and roots (ii) from *N. nimmoniana*.

**a** Strictosidine in *N. nimmoniana*, (i) leaves; (ii) roots.

**b** Strictosidinic acid in *N. nimmoniana*, (i) leaves; (ii) roots.

**Fig. S11. Phylogenetic relationship of DNA topoisomerase I in CPT-producing and non-producing species.** Three key amino acid mutations sites (colored rectangles) in CPT-producing plants are related to CPT-resistence. *O. pumila*, *O. liukiuensis,* and *C. acuminata* share N to S mutation (marked orange). *N*. *nimmoniana* and *C. acuminata* share specific N to K mutation (marked blue). *O. pumila* and *O. liukiuensis* share specific G to S mutation (marked green).

**Table S1.** **Relevant Compounds Detected in *O. pumila* plant and hairy root.**

| **Annotated Metabolite** | **Formula** | **Retention Time (min)** | **Calculated for [M+H]^+^** | **Found [M+H]^+^** | **Observed fragment Ion(s) in MS/MS (*m/z*)** |
| --- | --- | --- | --- | --- | --- |
| Tryptamine | C_10_H_12_N_2_ | 9.5 | 161.1073 | 161.1085 |  |
| Loganic acid | C_16_H_24_O_10_ | 7.5 (isomer1) 8.1 (isomer2) | 377.1442 | 377.1454 | 359, 215, 197, 179, 161, 151, 137, 133, 123, 109 |
| Loganin | C_17_H_26_O_10_ | 9.6 (isomer1) 11.0 (isomer2) | 391.1599 | 391.1599 | 371, 327, 283, 239, 195, 177, 133, 89 |
| Secologanic acid | C_16_H_22_O_10_ | 8.9 | 375.1286 | 375.1292 | 213, 195, 177, 151, 125, 109, 107, 95, 79, 77 |
| Secologanin | C_17_H_24_O_10_ | 8.9 | 389.1442 | 389.1459 | 227, 209, 195, 177, 165, 151, 149, 139, 109, 107 |
| Strictosidinic acid | C_26_H_32_N_2_O_9_ | 12.8 | 517.2181 | 517.219 | 500, 355, 338, 320, 269/268, 251, 194, 181/180, 170/168, 156, 151, 144, 125 |
| Strictosidine | C_27_H_34_N_2_O_9_ | 16.1 | 531.2337 | 531.2356 | 355, 335, 283, 237, 163, 133, 118 |
| Strictosamide | C_26_H_30_N_2_O_8_ | 24.9 | 499.2075 | 499.2083 | 337, 319, 267, 171, 144 |
| Strictosamide epoxide | C_26_H_30_N_2_O_9_ | 9.7 (isomer1) 11.9 (isomer2) 12.8 (isomer3) | 515.2024 | 515.2025 | 353, 335, 309, 283, 265, 238, 209, 184, 144 |
| Strictosamide diol | C_26_H_32_N_2_O_10_ | 9.7 | 533.2135 | 533.2134 | 371, 353, 283, 265, 160, 142, 132 |
| Strictosamide ketolactam | C_26_H_30_N_2_O_10_ | 11.8 (isomer1) 12.8 (isomer2) | 531.1973 | 531.2366 | 369, 351, 299, 281, 271, 253, 194, 176, 158 148, 130, 124, 106 |
| Pumiloside | C_26_H_28_N_2_O_9_ | 11.3 (isomer1) 12.3 (isomer2) | 513.1868 | 513.1885 | 351, 333, 315, 305, 281, 263, 235, 140 |
| Deoxypumiloside | C_26_H_28_N_2_O_8_ | 19.9 (isomer1) 22.4 (isomer2) | 497.1918 | 497.1931 | 335, 265, 247, 219, 183, 169, 142 |
| Camptothecin | C_20_H_16_N_2_O_4_ | 23.8 | 349.1183 | 349.1192 | 305, 277, 249, 219/220, 168 |
| Methoxycamptothecin | C_21_H_18_N_2_O_5_ | 25.5(isomer1) 26.7(isomer2) | 379.1288 | 379.1297 | 335, 325, 307, 292, 279, 264, 250, 235, 221, 211, 198, 156 |

**Table S2. Identification of candidate CPT biosynthetic pathway genes in *O. pumila* as revealed by sequence identity with characterized genes from the prestrictosidine biosynthetic pathways in *Catharanthus roseus*.**

| Gene | Protein (Cr) | gene ID (Op) | % (Op) | gene ID (Ca)* | % (Ca)* |
| --- | --- | --- | --- | --- | --- |
| DXS | ABI35993.1 | Op-4739.12578 | 76 | Cac_g024944.t1 | 78 |
| DXR | AAF65154.1 | Op-4739.66801 | 88 | Cac_g016318.t1 | 89 |
| CMS | ACI16377.1 | Op-4739.82921 | 78 | Cac_g018722.t1 | 78 |
| CMK | ABI35992.1 | Op-4739.39559 | 80 | Cac_g021688.t1 | 76 |
| MCS | AAF65155.1 | Op-4739.68299 | 77 | Cac_g008169.t1 | 74 |
| HDS | AAO24774.1 | Op-4739.52465 | 90 | Cac_g022763.t1 | 89 |
| HDR | ABI30631.1 | Op-4739.41122 | 84 | Cac_g014659.t1 | 84 |
| IPI | ABW98669.1 | Op-4739.44767 | 78 | Cac_g008847.t1 | 91 |
| GPPS | ACC77966.1 | Op-4739.61856 | 82 | Cac_g026508.t1 | 77 |
| G8H | CAC80883.1 | Op-4739.81921 | 82 | Cac_g017987.t1 | 77 |
| GOR | AHK60836.1 | Op-4739.44167 | 88 | Cac_g027560.t1 | 72 |
| ISY | AFW98981.1 | Op-4739.7966 | 79 | Cac_g006027.t1 | 66 |
| IO | AHK60833.1 | Op-4739.18892 | 90 | Cac_g032709.t1 | 78 |
| 7DLGT | BAO01109.1 | Op-7611.0 | 86 | Cac_g008744.t1 | 77 |
| 7DLH | AGX93062.1 | Op-4739.80257 | 84 | Cac_g012663.t1 | 70 |
| LAMT | ABW38009.1 | Op-4739.3983 | 78 | Cac_g005179.t1 | 54 |
| SLS | AAA33106.1 | Op-4739.35781 | 83 | Cac_g012666.t1 | 65 |
| STR | CAA43936.1 | Op-4739.20059 | 55 | Cac_g030447.t1 | 38 |
| SGD | AAF28800.1 | Op-4739.57459 | 48 | Cac_g024448.t1 | 51 |
| GS | AHK60846.1 | Op-4739.70992 | 58 | Cac_g002596.t1 | 58 |
| GO | AVM85916.1 | Op-4739.68050 | 44 | Cac_g004918.t1 | 38 |
| Redox1 | AVM85917.1 | Op-13708.0 | 71 | Cac_g002596.t1 | 66 |
| Redox2 | AVM85918.1 | Op-4739.1065 | 50 | Cac_g012543.t1 | 54 |
| SAT | AVM85919.1 | Op-1674.0 | 48 | Cac_g031644.t1 | 46 |
| PAS | AWJ76616.1 | Op-4739.86537 | 55 | Cac_g020090.t1 | 50 |
| DPAS | ANQ45231.1 | Op-5854.1 | 60 | Cac_g002596.t1 | 60 |
| TS | AVM85921.1 | Op-4739.18158 | 52 | Cac_g010448.t1 | 48 |
| CS | AVM85920.1 | Op-4739.18157 | 52 | Cac_g010448.t1 | 48 |

Only the top hit from the BLAST search is presented.

**Abbreviations:**

1-deoxy-D-xylulose 5-phosphate synthase 2 (DXS); 1-deoxy-D-xylulose-5-phosphate reductoisomerase (DXR); 4-diphosphocytidyl-methylerythritol 2-phosphate synthase (CMS); 4-diphosphocytidyl-2-C-methyl-D-erythritol kinase (CMK) ; 2C-methyl-D-erythritol 2,4-cyclodiphosphate synthase (MCS); GCPE protein (HDS); 1-hydroxy-2-methyl-butenyl 4-diphosphate reductase (HDR); Plastid isopentenyl pyrophosphate: dimethylallyl pyrophosphate isomerase (IPI); Geranyl pyrophosphate synthase (GPPS); Geraniol 8-hydroxylase (G8H); 8-hydroxygeraniol oxidoreductase (GOR); Iridoid synthase (ISY); Iridoid oxidase (IO); UDP-glucose iridoid glucosyltransferase (7DLGT); 7-deoxyloganic acid 7-hydroxylase (7DLH); loganic acid methyltransferase (LAMT); secologanin synthase (SLS); strictosidine synthase (STR); strictosidine beta-glucosidase (SGD); geissoschizine synthase (GS); geissoschizine oxidase (GO); stemmadenine O-acetyltransferase (SAT); precondylocarpine acetate synthase (PAS); dehydroprecondylocarpine acetate synthase (DPAS); tabersonine synthase (TS); tatharanthine synthase (CS)

**Table S3. Identities and similarities among STRs from *C. acuminata*, *N. nimmoniana*, and *O. pumila* used in this work.**

|  | **Identities (%)** | | | | | |
| --- | --- | --- | --- | --- | --- | --- |
| **Similarities (%)** |  | *Ca*STR1 | *Ca*STR2 | *Ca*STR3 | *Op*STR | *Nn*STR |
|  | *Ca*STR1 | 100 | 96.626 | 65.244 | 41.159 | 34.743 |
|  | *Ca*STR2 | 99.08 | 100 | 65.549 | 40.549 | 34.441 |
|  | *Ca*STR3 | 84.756 | 84.756 | 100 | 40.729 | 33.033 |
|  | *Op*STR | 72.561 | 72.561 | 72.948 | 100 | 34.036 |
|  | *Nn*STR | 64.35 | 64.048 | 62.162 | 67.169 | 100 |

**Table S4** Kinetic parameters of *Op*STR towards secologanin and secologanic acid.

| Substrates | K_m_ (mM) | V_max_ (nkat mg^−1^) | K_cat_ (min^-1^) | K_cat_/K_m_ (min^-1^mM^-1^) |
| --- | --- | --- | --- | --- |
| secologanin | 0.382±0.0681 | 0.6288±0.0267 | 3.144 | 8.23 |
| secologanic acid | 0.9214±0.3097 | 0.03667±0.0046 | 0.009168 | 0.00995 |

**Table S5. Primers list used in this study.**

| **Primer Name** | **Sequence** |
| --- | --- |
| *Op*STR-BamHI-F | CGCGGATCCATGGAGTTCTTCGAATTTATTGAAGCACCA |
| *Op*STR-SalI-R | ACGCGTCGACCTAGAAAGAAGAAAATTCCTTGAATGATGCATTGAG |
| *Op*TDC-BamHI-F | CGCGGATCCATGGGCAGCATTAGT |
| *Op*TDC-SalI-R | ACGCGTCGACTTACTCAATGATATTGGTTTTCG |
| *Op*LAMT-BamHI-F | CCATGGCTGATATCGGATCCATGGCCCCAACCATGGACA |
| *Op*LAMT-Sal-R | GCGGCCGCAAGCTTGTCGACTTAATTGATTTTGCGTTTTAGGATAAGAAATAGAT |
| *Ca*STR1-BamHI-F | CGGGATCCATGCGCAATTCATTCCATATCGTCAACTGCA |
| *Ca*STR1-HindIII-F | CCCAAGCTTTTAACGCTTGTAAACACCAACAAACTTTGGATATA |
| *Ca*STR2-BamHI-F | CGGGATCCATGGCAATTGTCTGGAGTACTTCCAAAACTAGT |
| *Ca*STR2-HindIII-F | CCCAAGCTTTTAACGCTTGTAAACACCAACAAACTTTGGATATAGAGA |
| *Ca*STR3-BamHI-F | CGGGATCCATGGCAATTCTCAAGAGTTCCAGAACTAGT |
| *Ca*STR3-HindIII-F | CCCAAGCTTTTATGGCTTGTAAACACCAACAAAGTTCGGA |
| *Op*SLS1-SpeI-F | TAAAGGGCGGCCGCACTAGTATGGAGATGGATATTATCATCCGTCAGGC |
| *Op*SLS1-PacI-R | GGCGAAGAATTGTTAATTAATCACTCGAGTTTGCGGTAGATCAC |
| *Op*SLS2-SpeI-F | TAAAGGGCGGCCGCACTAGTATGGAGATGGATACAATTATGCAGAAAGCC |
| *Op*SLS2-PacI-R | GGCGAAGAATTGTTAATTAATTATTTTATTATAGGTTCTGCAAGTTGATATTCTAG |
| *Op*SLS3-SpeI-F | TAAAGGGCGGCCGCACTAGTATGGAGATGGATATTATACTTGAGGCAATTGC |
| *Op*SLS3-PacI-R | GGCGAAGAATTGTTAATTAATCAAAGCTTGCGGAAGATGACAG |
| *Op*SLS4-NotI-F | CCTCACTAAAGGGCGGCCGCATGCAAACATCCTACGGTTTGACAG |
| *Op*SLS4-PacI-R | GGCGAAGAATTGTTAATTAATTACAGTTTGTGCAAAATCAAGTGAGCAC |
| *Op*SLS5-NotI-F | CCTCACTAAAGGGCGGCCGCATGTTAGGAATGGAAGTGAACCTCAATTCA |
| *Op*SLS5-PacI-R | GGCGAAGAATTGTTAATTAATTAGAGTTTCTGCAATATTAAGTGAGCACCAA |
| *Op*SLS6-SpeI-F | TAAAGGGCGGCCGCACTAGTATGGAAAATCTCTATAGCTTAGTTGGAGTTTTG |
| *Op*SLS6-PacI-R | GGCGAAGAATTGTTAATTAACTACAGTTTGCGCAAAATTAACTGAGCA |
