# Supplementary figures and images for: Divergent camptothecin biosynthetic pathway in Ophiorrhiza pumila
Source: BMC Biol. 2021 Jun 16;19:122. doi: 10.1186/s12915-021-01051-y (PMC8207662; doi:10.1186/s12915-021-01051-y)

**A**

M *Op*TDC M *Op*LAMT

75 kDa  
65 kDa  
45 kDa  
35 kDa  
25 kDa  
15 kDa

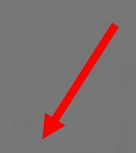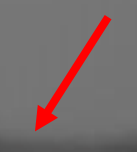

**B**

M *Op*STR *Op*STR *Op*STR

75 kDa  
65 kDa  
45 kDa  
35 kDa  
25 kDa  
15 kDa

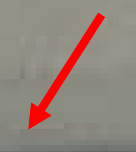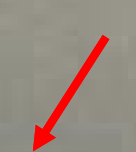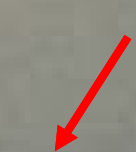

Supplement: Supplementary file 2 — Additional file 2:. The original, uncropped SDS-PAGE of Fig. S6. [file 12915_2021_1051_MOESM2_ESM.pdf]
